# Novel evidence on sepsis-inducing pathogens: from laboratory to bedside

**DOI:** 10.3389/fmicb.2023.1198200

**Published:** 2023-06-23

**Authors:** Sebastian Gatica, Brandon Fuentes, Elizabeth Rivera-Asín, Paula Ramírez-Céspedes, Javiera Sepúlveda-Alfaro, Eduardo A. Catalán, Susan M. Bueno, Alexis M. Kalergis, Felipe Simon, Claudia A. Riedel, Felipe Melo-Gonzalez

**Affiliations:** ^1^Facultad de Ciencias de la Vida, Universidad Andres Bello, Santiago, Chile; ^2^Millennium Institute on Immunology and Immunotherapy, Santiago, Chile; ^3^Departamento de Genética Molecular y Microbiología, Facultad de Ciencias Biológicas, Millennium Institute on Immunology and Immunotherapy, Pontificia Universidad Católica de Chile, Santiago, Chile; ^4^Departamento de Endocrinología, Facultad de Medicina, Pontificia Universidad Católica de Chile, Santiago, Chile

**Keywords:** sepsis, inflammation, immunology, microorganisms, diagnostics, prognosis, therapy

## Abstract

Sepsis is a life-threatening condition and a significant cause of preventable morbidity and mortality globally. Among the leading causative agents of sepsis are bacterial pathogens *Escherichia coli*, *Klebsiella pneumoniae*, *Staphylococcus aureus*, *Pseudomonas aeruginosa*, and *Streptococcus pyogenes*, along with fungal pathogens of the *Candida* species. Here, we focus on evidence from human studies but also include *in vitro* and *in vivo* cellular and molecular evidence, exploring how bacterial and fungal pathogens are associated with bloodstream infection and sepsis. This review presents a narrative update on pathogen epidemiology, virulence factors, host factors of susceptibility, mechanisms of immunomodulation, current therapies, antibiotic resistance, and opportunities for diagnosis, prognosis, and therapeutics, through the perspective of bloodstream infection and sepsis. A list of curated novel host and pathogen factors, diagnostic and prognostic markers, and potential therapeutical targets to tackle sepsis from the research laboratory is presented. Further, we discuss the complex nature of sepsis depending on the sepsis-inducing pathogen and host susceptibility, the more common strains associated with severe pathology and how these aspects may impact in the management of the clinical presentation of sepsis.

## 1. Introduction

Sepsis, a life-threatening organ dysfunction caused by a dysregulated host response to infection (Singer et al., 2016), is a significant cause of geriatric, maternal, neonatal, and child mortality. The World Health Organization (WHO) responded in 2017 with a resolution acknowledging sepsis as a major cause of preventable morbidity and mortality globally, while highlighting some of the most frequent pathogens connected etiologically to this condition, their primary site of infection, the increasing contribution of nosocomial infections, and their alarming resistance to antibiotics [World Health Organization (WHO), 2017]. Great strides toward an accurate quantification of its incidence and mortality (Rudd et al., 2020), and the pathogens accounting for its excess of mortality (GBD 2019 Antimicrobial Resistance Collaborators, 2022), have been made since that resolution. While epidemiological findings raise awareness and aid in the shaping of public health policies, molecular discoveries contribute to the identification of host factors of susceptibility and pathogen mechanisms of immune evasion, structuring the therapeutic tools of the future.

Bacterial pathogens lead cases of bloodstream infections (BSI; Gouel-Cheron et al., 2022) with *Escherichia coli*, *Klebsiella pneumoniae*, *Staphylococcus aureus*, *Pseudomonas aeruginosa*, and *Streptococcus pyogenes*, as the as the pathogens with the largest number of attributable deaths (Diekema et al., 2019; GBD 2019 Antimicrobial Resistance Collaborators, 2022). Among fungal pathogens, *Candida* species are the most frequent pathogens in the critical care setting, with strong nosocomial association (Delaloye and Calandra, 2014; Gouel-Cheron et al., 2022). Viral agents are less frequently regarded as the cause of sepsis and are often deemed as facilitators to infection by secondary agents (Lin et al., 2018). Nevertheless, Herpes simplex virus, Enterovirus, Influenza, Adenovirus, Dengue virus, and recently the novel severe acute respiratory syndrome coronavirus 2 (SARS-CoV-2), are considered as viral causes of sepsis (Shane et al., 2017; Teparrukkul et al., 2017; Iuliano et al., 2018; Alhazzani et al., 2020).

Because only a fraction of the articles included in this work regarded the comprehensive clinical continuum that sepsis represents, a pragmatic approach translating results from invasive infection and progression into the bloodstream, either by extension or by analogy, was adopted. Thus, this review summarizes recent findings in terms of pathogen epidemiology, virulence factors, host factors of susceptibility, mechanisms of immunomodulation, current therapies, antibiotic resistance, and opportunities for diagnosis, prognosis, and therapeutics, focusing on evidence from human studies but also including cellular and molecular evidence from *in vitro* and *in vivo* studies, all through the looking glass of BSI and sepsis.

## 2. Escherichia coli

*Escherichia coli* is a Gram-negative bacillus with remarkable phylogenetic diversity. Although mostly recognized for its pathogenic role in diarrheal diseases, and less frequently to extraintestinal illness, *E. coli* is also a part of the commensal microbiome of the host. Commensal strains (originating typically from phylogroup A) rarely cause diseases in healthy hosts, as they lack specialized virulence traits. Intestinal-pathogenic *E. coli* comprise groups diarrheagenic, enteropathogenic, enterohemorrhagic, enterotoxigenic, enteroaggregative, enteroinvasive, and diffusely adherent (originating typically from phylogroups B1 and E; Kaper et al., 2004; Bachmann et al., 2015). Extraintestinal *E. coli* (ExPEC) comprise a growing group that includes uropathogenic *E. coli* (UPEC), sepsis-associated *E. coli* (SEPEC), and neonatal meningitis *E. coli* (NMEC), among others (Desvaux et al., 2020), originating typically from phylogroups B2 and occasionally from phylogroups D, F, or G (Escobar-Páramo et al., 2004; Clermont et al., 2019). In fact, a recent prospective observational cohort study on *E. coli* bacteremia found that phylogroups most frequently associated with fatal outcome after 28 days were, in order, B2 (46%), D (17%), and B1 (15%; de Lastours et al., 2020). The main source of *E. coli* bacteremia is urinary tract infection (representing more than 50% of the cases; Bonten et al., 2020), with advanced age (>65 years) representing the greatest risk factor for asymptomatic *E. coli* bacteremia of urinary source (OR = 1.8–2.95; Bai et al., 2020). Consistently, UPEC isolated from patients with pyelonephritis exhibit much higher serum resistance (82–93%) than fecal *E. coli* isolates (57%; Coggon et al., 2018; Table 1). Although widely considered as the leading pathogen for bacteremia (Shorr et al., 2006; Al-Hasan et al., 2012), the predominance of *E. coli* depends on the timeframe, geographical location, and age of the patients included in the analysis.

**Table 1:** Summary of novel diagnostic/prognostic markers and resistance factors of interest for sepsis detailed in this review.

Organism	Marker/factor	References
Resistance factors		
*E. coli*	UPEC bacteremia	Coggon et al. (2018)
*K. pneumoniae*	Serotype O1, O2, or O3 bacteremia	Choi et al. (2020)
*S. aureus*	PVL^+^ MRSA bacteremia	Zhao et al. (2022)
*P. aeruginosa*	Resistance genes *blaGES*, *aadB*, *gyrA* (T83I), and *parC* (S87L)	Recio et al. (2021)
*S. pyogenes*	*emm43.4*/PBP2x-T553K or emm93.0 bacteremia	Hayes et al. (2020) and Ron et al. (2022)
Novel diagnostic/prognostic markers		
*S. aureus*	Measurement of genes *COX7C*, *NDUFA4*, *ATP5J*, *NDUFB3*, and *COX7A2*	Wu H. et al. (2021)
	Measurement of m6A-SNPs	Sun et al. (2020)
	Measurement of caspase-1, IL-18, and NLRP3	Rasmussen et al. (2019)
	Measurement of C5a and IL-10	Eichenberger et al. (2020)
	Measurement of neutrophil/lymphocyte ratio	Greenberg et al. (2018)
*S. pyogenes*	Multidimensional scaling of leukocyte and platelet abundance	Loof et al. (2018)
*Candida* spp.	Machine-learning	Ripoli et al. (2020)
	Delta neutrophil index	Park et al. (2020)
	Multivariate risk score	Poissy et al. (2020)
	Composite SOFA/CCI score	Asai et al. (2021)
	Prior corticosteroid use	Kayaaslan et al. (2021)

ATP5J, ATP synthase-coupling factor 6; C5a, complement component 5a; COX7A2, cytochrome c oxidase subunit 7A2; COX7C, cytochrome c oxidase subunit 7C; IL-10, interleukin 10; IL-18, interleukin 18; m6A-SNPs, N6-methyladenosine associated single-nucleotide polymorphisms; MRSA, methicillin-resistant *S. aureus*; NDUFA4, cytochrome c oxidase subunit NDUFA4; NDUFB3, NADH dehydrogenase [ubiquinone] 1 beta subcomplex subunit 3; NLRP 3, NOD-like receptor family pyrin domain-containing 3; PVL, panton–valentine leukocidin; UPEC, uropathogenic *E. coli*; VEGF, vascular endothelial growth factor.

### 2.1. Epidemiology

The SENTRY program for global antimicrobial surveillance places *E. coli* as the number one pathogen causing BSI worldwide since 2005 (Diekema et al., 2019). However, this is not consistent in the United States, where *S. aureus* leads the ranking since 1997. In fact, a recent report on the epidemiology of hospital-acquired BSI in intensive care unit patients in Europe reveals that *Klebsiella* spp. lead the ranking of Gram-negative bacteremia, relegating *E. coli* to the second place (Tabah et al., 2023). Similarly, data from a prospective observational study conducted in Thailand, which included data from 3,806 patients, found that *E. coli* was the most frequent causative agent for BSI, and that age over 70 years carries an increased hazard ratio of 1.5 for 28-day mortality (Somayaji et al., 2021). A recent meta-analysis reported that the incidence of *E. coli* bacteremia is increasing over time in select high-income countries around the globe with pooled mortality rates ranging from 10.7 to 14.3% (Bonten et al., 2020). Also, data from the SENTRY program place *E. coli* as the leading pathogen causing BSI only in ages older than 64 years old, while for the rest of the age groups *S. aureus* leads the ranking, a fact that was ratified in a 10-year report on the epidemiology of bacteremia in a Portuguese pediatric population (Ferreira et al., 2023). Nevertheless, a recent cohort study representative of the United States, which analyzed 217,480 neonatal patients, found an overwhelming majority of *E. coli*-associated sepsis (Stoll et al., 2020). Remarkably, *E. coli* dominance in hospital onset bacteremia (Diekema et al., 2019) is significantly diluted during concomitant viral co-infection (Glass et al., 2022), especially in patients hospitalized for coronavirus disease 2019 (COVID-19; Garcia-Vidal et al., 2021).

### 2.2. Pathogen factors

Virulence factor diversity for *E. coli* isolated from blood comprises genes coding for proteins related to adherence (*ecp*, *hcp*, *eaeH*, *fim*, and *pap*), intracellular traffic (*upaG*, *ehaB*, and *agn43*), invasion (*tia*), iron uptake (*sitA*, *chuA*, *fyuA*, and *iuccC*), and toxins (*hlyE*/*clyA*, *usp.,* and *senB*) allowing bacteria to reach and survive in the bloodstream (Kim et al., 2022). However, ExPEC makes use of additional virulence traits, members of the serine protease autotransporters of Enterobacteriaceae (SPATE) superfamily involved in evasion from host immune defense mechanisms (Abreu et al., 2015). A recent prospective observational cohort study analyzing blood isolates from 278 patients found SPATE coding genes in 61% of the isolates, with an overwhelming presence in phylogroup B2, with *sat* and *vat* as the most prevalent of them (Freire et al., 2020). SPATE gene *sat* is a class 1-SPATE recognized as a cytotoxic factor with proteolytic activity (Freire et al., 2022) and *vat* is a class 2-SPATE cytotoxic factor (Nichols et al., 2016; Díaz et al., 2020), and both displayed a significant presence in ExPEC isolates of phylogroups B2, D, E, and F (Freire et al., 2020). *In vitro* evidence on virulence factors reveals that *E. coli* adheres to endothelial cells by a direct and necessary interaction between bacterial cell membrane protein OmpA and endothelial integrin αVβ3 which, in turn, activates endothelial cells via calcium-dependent intracellular cascades that ultimately lead to a downregulation of VE-Cadherin (Tapia et al., 2019), increased vascular permeability (Gatica et al., 2020), and endothelial apoptosis (McHale et al., 2018). Interestingly, all these readouts displayed not different to uninfected controls when using OmpA-deficient mutant strains, strongly underlining the role of OmpA in the genesis of sepsis at the cellular level. A summary of the factors detailed in this section is listed in Table 2.

**Table 2:** Summary of novel pathogen factors of interest for sepsis detailed in this review.

Organism	Activity	Pathogen factor	References
*E. coli*	Survival	*iss*	Fröding et al. (2020)
	Adhesion	*iha17, ecp, hcp, eaeH, fim,* and *pap*	de Lastours et al. (2020) and Kim et al. (2022)
	Intracellular traffic	*upaG*, *ehaB*, and *agn43*	
	Invasion	*tia*	
	Iron metabolism	*sitA*, *chuA*, *fyuA*, and *iuccC*	
	Toxins	*hlyE*/*clyA*, *usp,* and *senB*	
	Cytotoxicity	SPATE coding genes *sat* and *vat*	Freire et al. (2020)
	Cell infection	*ompA*	McHale et al. (2018)
*K. pneumoniae*	Invasion	*rmpA/2*	Cienfuegos-Gallet et al. (2022), Kochan et al. (2022), and Liao et al. (2022)
	Iron metabolism	*ybt,* iucA, and *iroB*	
	Cytotoxicity	*clb*	
	Metabolism	*peg-344*	
*S. aureus*	Survival	*capA*	Recker et al. (2017)
	Cytotoxicity	PVL, TSST-1, and *hly*	Ahmad et al. (2020), Monecke et al. (2020), and Sun et al. (2021)
*P. aeruginosa*	Survival	*mifR*	Xiong et al. (2022)
	Invasion	*hepP*	Dzvova et al. (2018)
	Iron metabolism	Hxu, Has, and Phu systems	Otero-Asman et al. (2019) and Yang F. et al. (2022)
	Cytotoxicity	*exoS*	Recio et al. (2021)
*S. pyogenes*	Invasion	*emm*, *speG*, *speH*, *speJ*, and *speK*	Imöhl et al. (2017) and Sánchez-Encinales et al. (2019)
	Metabolism	*spy1476*, *spy1343*	Sitkiewicz and Musser (2017) and Kant and Pancholi (2021)

PVL, panton–valentine leukocidin; SPATE, serine protease autotransporters of Enterobacteriaceae; and TSST-1, toxic shock syndrome toxin-1.

### 2.3. Host factors

Clinical manifestations of *E. coli* bacteremia include fever, disorientation, hypotension, and respiratory failure, and may include septic shock, which is estimated to be present in about 25% of bacteremic patients (Kang et al., 2005). In fact, a recent prospective observational cohort study found a significant association between *E. coli* bacteremia and adverse outcomes after 28 days for the following clinical presentations: cancer, chronic peripheral arteritis, sepsis, and septic shock at initial presentation, infection of the digestive tract and airway, and start of adequate antibiotic therapy after 48 h of onset bacteremia (de Lastours et al., 2020). Interestingly, bacteremias starting as urinary tract infections displayed a greater strength of association with the survivor group, representing a noteworthy point of inflection for these types of infections. The latter is not an isolated figure, as confirmed in a multivariate analysis from a recent retrospective cohort study on *E. coli* bacteremia, where this observation urinary tract infection is reported as a remarkable protective factor (OR = 0.07) for the outcome 30-day mortality (Chapelet et al., 2017). Furthermore, multivariate analysis reveals an astounding OR of 6.54 for pulmonary portal of entry as determinant of 28-day mortality, after adjustment. Other determinants included in the final multivariate model were infection with bacterial factor STc88 (OR = 3.62), expression of virulence factor iha_17 (OR = 4.41), and other comorbidities (OR = 1.14; de Lastours et al., 2020). Similarly, another prospective observational cohort study found a significant association between *E. coli* bacteremia and septic shock or death within 72 h in patients presenting hematologic cancer or history of transplantation (OR = 16.34), reduced daily living activity (OR = 3.85), and presence of virulence factor *iss* (OR = 7.71), after adjustment in a multivariate analysis (Fröding et al., 2020). The excess risk revealed for oncologic, or transplantation patients is a call for caution to practitioners attending such conditions. A summary of the factors detailed in this section is listed in Table 3.

**Table 3:** Summary of novel risk and protective factors of interest for sepsis detailed in this review.

Organism	Factor	References
Pathogen risk factors		
*E. coli*	Presence of phylogroups B2, D, and B1	de Lastours et al. (2020)
*K. pneumoniae*	Presence serotypes K1, K2, K20, K54, K57	Liao et al. (2022)
	Presence of *bla*KPC-bearing strains	Hu et al. (2021)
*S. aureus*	Presence of PVL^+^ MRSA strains	Imauven et al. (2022)
*P. aeruginosa*	Presence of MLST, ST235 or O11 serotype	Recio et al. (2021)
	Strong biofilm producing strains	di Domenico et al. (2021)
	Non-motile strains	Gupte et al. (2021)
*Candida* spp.	Presence of BDG^+^ species	Agnelli et al. (2019)
Host risk factors		
*E. coli*	Advanced age	Bai et al. (2020) and Somayaji et al. (2021)
	Neonate infection	Stoll et al. (2020)
	Chronic comorbidities: cancer, chronic peripheral arteritis	de Lastours et al. (2020)
	Bacteremia with pulmonary portal of entry	Chapelet et al. (2017)
*K. pneumoniae*	Bacteremia with pulmonary portal of entry	Chen I. R. et al. (2022)
	Presence of central venous catheter	Ang et al. (2022)
*S. aureus*	Advanced age, male sex	Bassetti et al. (2018) and Imam et al. (2019)
	Presence of genes *COX7C, NDUFA4, ATP5J, NDUFB3*, and *COX7A2*	Wu H. et al. (2021)
*P. aeruginosa*	Advanced age, male sex	Esparcia et al. (2019)
	Indwelling urinary catheter	Esparcia et al. (2019) and Tan et al. (2021)
	Long-term hospital stay	Tan et al. (2021)
	Immunocompromise	Hammer et al. (2017)
	Bacteremia with pulmonary portal of entry	
	X-linked agammaglobulinemia	Bhardwaj et al. (2017), Biscaye et al. (2017), Birlutiu et al. (2019), and Huang H. et al. (2020)
*S. pyogenes*	Low levels of VEGF	Lu et al. (2022)
*Candida* spp.	Concomitant bacteremia	Lee et al. (2020), Pieralli et al. (2021), Zhong et al. (2022), and Gebremicael et al. (2023)
	SOFA score	Bienvenu et al. (2020), Jung et al. (2020), Huang H. Y. et al. (2020), and Kutlu et al. (2022)
	CVC	Lee et al. (2020) and Huang H. Y. et al. (2020)
	Liver cirrhosis	González-Lara et al. (2017), Battistolo et al. (2021), and Meyahnwi et al. (2022)
	Kidney dysfunction	Poissy et al. (2020), Mazzanti et al. (2021), and Kutlu et al. (2022)
	Charlson Comorbidity Index ≥4	Bassetti et al. (2020), Yoo et al. (2020), and Kim et al. (2021)
	Concomitant neoplasia	Lee et al. (2020), Battistolo et al. (2021), and Vázquez-Olvera et al. (2023)
	Current azole therapy	Lee et al. (2020)
	Age ≥ 65	Meyahnwi et al. (2022)
	Concurrent antibiotic therapy	
	Neutropenia	Kim et al. (2021)
	Total parenteral nutrition	Pieralli et al. (2021) and Kutlu et al. (2022)
	Hemodialysis	Bassetti et al. (2020)
	Cardiovascular surgery	Mazzanti et al. (2021)
	IV catheter	Huang H. Y. et al. (2020)
	MODS ≥6	Chen et al. (2020) and Yoo et al. (2020)
	Concomitant severe sepsis	González-Lara et al. (2017)
	Required vasopressor therapy	Gebremicael et al. (2023)
	Liver dysfunction	
	Broad-spectrum antibiotic use before candidemia	Kutlu et al. (2022)
	Thrombocytopenia	
	Delayed treatment	Bienvenu et al. (2020)
Host protective factors		
*E. coli*	Bacteremia with urinary portal of entry	Chapelet et al. (2017) and de Lastours et al. (2020)
*P. aeruginosa*	Levels of hemoglobin in pediatrics	Kung et al. (2020)
*S. pyogenes*	VEGF	Lu et al. (2022)
	Endosomal TLR13 pathogen recognition	Hafner et al. (2019)
*Candida* spp.	CVC removal	Kutlu et al. (2022)

ATP5J, ATP synthase-coupling factor 6; BDG, (1,3)-β-D-Glucan; COX7A2, cytochrome c oxidase subunit 7A2; COX7C, cytochrome c oxidase subunit 7C; CVC, central venous catheter; GI disease, gastrointestinal disease; blaKPC, b-lactamase *K. pneumoniae*; MLST, multilocus sequence typing; MODS, multiple organ dysfunction score; MRSA, methicillin-resistant *S. aureus*; NDUFA4, cytochrome c oxidase subunit NDUFA4; NDUFB3, NADH dehydrogenase [ubiquinone] 1 beta subcomplex subunit 3; PVL, panton–valentine leukocidin; SOFA, sepsis organ failure assessment; TLR13, toll-like receptor 13; VEGF, vascular endothelial growth factor.

### 2.4. Treatment

Empiric antimicrobial therapy follows a general Gram-negative bacteremia algorithm with prolonged delivery of broad-spectrum β-lactam antibiotics as the first indication (Evans et al., 2021). Directed therapy is aimed at restraining resistance with recommendations to switch to a single agent with the narrowest spectrum to which the organism is susceptible. A recent prospective observational cohort study found that amoxicillin/clavulanic acid, cotrimoxazole, and fluoroquinolone, had the strongest association between antibiotic resistance and 28-day mortality in *E. coli* bacteremia (de Lastours et al., 2020). Favorably, none of the non-survivors presented any carbapenem resistance, which was present in only one out of 493 survivors. Moreover, multivariate analysis reveals an increased risk for 28-day mortality by broad-spectrum β-lactam and/or third-generation cephalosporin resistant strains in patients presenting chronic alcoholism (OR = 3.04), initiating adequate antibiotic treatment after 48 h (OR = 3.04), or with a history of bacteremia (OR = 2.81), after adjustment (de Lastours et al., 2020). According to the latest global report on antimicrobial resistance by the WHO, the median resistance to third generation cephalosporins in *E. coli* bloodstream confirmed infections is 41.8% [World Health Organization (WHO), 2022a]. It is suggested that cefotaxime and ceftriaxone are the most suitable antibiotics to monitor cephalosporin resistance in *E. coli* BSI. Thus, pathogen-directed analyses are key to guide the development and implementation of strategies on age-targeted prevention, antimicrobial resistance, and new therapies.

## 3. Klebsiella pneumoniae

*Klebsiella pneumoniae* is a Gram-negative, encapsulated, non-motile, facultatively anaerobic bacterium. *Klebsiella* species are commonly found in soil, water, plants, and livestock (Morgado et al., 2022), and *K. pneumoniae* in human hosts is not strictly pathogenic. In fact, *K. pneumoniae* colonizes the nasal and digestive tract without causing any symptomatic disease (Chang et al., 2021), and it is proposed that gastrointestinal colonization serves as a major reservoir for transmission and infection to other sites (Martin et al., 2016). Moreover, studies show a high level of association between gastrointestinal carriage and subsequent infection by a patient’s own *K. pneumoniae* strains (Martin et al., 2016; Gorrie et al., 2017). Although the exact mechanisms are unclear, *K. pneumoniae* progression from intestinal territories has been related to a disproportion in bacterial density of colonizing strains, especially when host defenses are challenged, e.g., during cancer, diabetes mellitus, and alcohol abuse (Happel and Nelson, 2005). Traditionally, serotypes of *Klebsiella* isolates have been identified and followed using typing antisera. Different O and K serotypes are produced as a result of the detection of distinctive variants of surface-exposed polysaccharides, named O-antigens and K-antigens, by certain antibodies. O-antigens constitute the outer layer of lipopolysaccharide (LPS), whereas K-antigens are a component of the bacterial capsule polysaccharide (CPS). To date, eight serotypes for O-antigens and 77 for K-antigens have been described (Follador et al., 2016).

### 3.1. Epidemiology

A recent multi-country collection study revealed that *K. pneumoniae* geographical diversity is dominated worldwide by antigen O1, followed by antigen O2, which displayed a larger proportion in Europe, and antigen O3, less represented in Africa (Choi et al., 2020). Additionally, a recent report on worldwide BSI identified *K. pneumoniae* as the third most prevalent pathogen consistently over the last 25 years (Diekema et al., 2019). Moreover, various publications on *K. pneumoniae* progression into the bloodstream identified pulmonary, abdominal, and urinary sites as the leading sources of infection (Togawa et al., 2015; Hyun et al., 2018; Juan et al., 2019; Huang Y. T. et al., 2020; Li M. et al., 2023). Interestingly, all these reports are successful in identifying the primary source of infection, a fact that differs from clinical findings reported before 2010, in which *K. pneumoniae* bacteremia of unknown origin reached up to 58% of cases (Tsay et al., 2002; Tumbarello et al., 2006). Among the most widely distributed *K. pneumoniae*, serotype antibiotic resistance was predominantly associated to serotypes carrying antigens O2 and O3, while serotypes carrying antigen O1 (the most frequently distributed worldwide) was associated with sensitivity to extended spectrum cephalosporins, fluoroquinolone, and carbapenems, among many other antibiotics (Choi et al., 2020; Table 1). According to the latest global report on antimicrobial resistance by the WHO, *K. pneumoniae* is the third leading agent causing bacteremia with an increasing resistance to third- and fourth-generation cephalosporin and co-trimoxazole therapy [World Health Organization (WHO), 2022a], pushing the use of carbapenems (Souli et al., 2017).

### 3.2. Pathogen factors

Among the most prevalent virulence factors identified in BSI in the United States are genes *ybt*, *clb*, *iucA*, *rmpA*, *rmpA2*, and *iroB*, of which, *iucA* and *rmpA/2* have been associated with carbapenem resistance (Kochan et al., 2022). Consistently, *iucA*, *rmpA*, *rmpA2*, *iroB*, and *peg-344*, were recently described as the most prevalent virulence genes in Taiwan (Liao et al., 2022), while genes *ybt*, *iuc*, and *rmp* were recently described as the most prevalent in China (Cienfuegos-Gallet et al., 2022). Co-infection of *K. pneumoniae* with other bacterial agents (i.e., *Acinetobacter baumannii*, *P. aeruginosa*, and *E. coli*, among other less frequent bacteria) has been documented and sized previously (Karakonstantis et al., 2022; Lv et al., 2022).

### 3.3. Host factors

Multivariate analyses report nosocomial pneumonia, high SOFA score, inappropriate treatment (Chen I. R. et al., 2022), high APACHE II score, development of septic shock (Wu X. et al., 2021), and central venous catheter (Ang et al., 2022), as significant risk factors for mortality from *K. pneumoniae* BSI. Moreover, BSI with serotypes K1, K2, K20, K54, and K57, has also been found associated to increased mortality distributions (Liao et al., 2022). Furthermore, infection with *K. pneumoniae* β-lactamase (*bla*_KPC_)-harboring strains has been found as an independent risk factor for mortality (Hu et al., 2021). Co-infection of *K. pneumoniae* with other viral agents, particularly with SARS-CoV-2, has been well described (Damico et al., 2022; Said et al., 2022), reviewed (Santos et al., 2022), and sized (Lansbury et al., 2020; Kariyawasam et al., 2022; Santos et al., 2022) elsewhere. Of note, the proportion of *K. pneumoniae* isolates from COVID-19 patients has been documented to be significantly lower compared to COVID-negative controls, implicating that infection with viral agents may reduce the chance to develop bacteremia, a subject that requires further research (Glass et al., 2022). A summary of the factors detailed in this section is listed in Table 2.

### 3.4. Treatment

In line with general Gram-negative bacteremia treatment, empiric antimicrobial therapy considers prolonged delivery of broad-spectrum β-lactam antibiotics as the first indication (Evans et al., 2021). However, driven by the extensive use of carbapenems, the growth and spread of carbapenem-resistant bacterial infections has emerged as a major public health concern in recent decades. In fact, *K. pneumoniae* resistance to carbapenems has experienced a 3-fold increase worldwide since 2016 [World Health Organization (WHO), 2022a]. A wise strategy to overcome this new threat is founded on the use of carbapenem antibiotics in combination with β-lactamase inhibitors, such as clavulanic acid, sulbactam, and tazobactam. Thus, notorious advances in terms of drug efficacy and safety have been made over the last decade with new β-lactam/β-lactamase inhibitor combinations (BLIC) synergistically restoring antimicrobial sensitivity, e.g., aztreonam/avibactam, ceftazidime/avibactam, imipenem/relebactam, meropenem/vaborbactam, and cefepime/zidebactam, among others (Vázquez-Ucha et al., 2020).

A recent study in Taiwan reports a significant restoration of imipenem activity against carbapenem-nonsusceptible *K. pneumoniae* when combined with relebactam, increasing susceptibility from 0.8 to 88.4%, 21.4 to 42.9%, and 30 to 40%, in isolates producing carbapenemases of Ambler classes A, B, and D, respectively (Yang T. Y. et al., 2022). In accordance, regarding the same combination, a study analyzing *K. pneumoniae* isolates from Spain and Portugal reports a major decrease in resistance in Ambler class A carbapenemase-harboring strains, while reporting no resistance to ceftazidime/avibactam combination in isolates producing carbapenemases of Ambler classes A and D (Hernández-García et al., 2022). In fact, experimental combination of ceftazidime/avibactam and aztreonam (Shah et al., 2021) or possibly meropenem (Parruti et al., 2019) has been reported successfully not only in clinical case reports but also in a recent cohort study (adjusted HR = 0.136; Zheng et al., 2021). Additionally, use of fosfomycin in combination with third- and fourth-generation cephalosporin has been thoroughly (in a retrospective cohort study analyzing 104 cases of carbapenem resistant *K. pneumoniae* bacteremia) demonstrated as protective (adjusted OR = 0.07) against mortality by sepsis (Liao et al., 2017). While these promising results constitute the latest evidence on advanced approaches in patients, further evidence from animal models reveals that rifampin alone or in combination with colistin have the strongest effect against carbapenemase-producing *K. pneumoniae* sepsis mortality *in vivo* (Pachón-Ibáñez et al., 2018). Moreover, use of amikacin alone or in combination with fosfomycin significantly reduced circulating bacterial load in a sepsis model using carbapenemase-producing *K. pneumoniae* strains (Cebrero-Cangueiro et al., 2021).

In the quest for new therapies, a recent report on animal *K. pneumoniae* sepsis shows that the use of mushroom-derived β-glucans are able to reduce bacterial load while improving physiological parameters associated to the pathobiology of sepsis (i.e., arterial pO_2_, plasma lactate, pulmonary compliance, and arterial alveolar oxygen gradient; Masterson et al., 2020). In line with this, Bergenin monohydrate (a plant extract with immunomodulatory properties) has been described to reduce reactive oxygen species (ROS) production and increase cell viability *in vitro* while increasing levels of superoxide dismutase (SOD) and GSH, and reducing bacterial load, levels of inflammatory cytokines interleukin (IL)-6, IL-1β, prostaglandin E2 (PGE2), and tumor necrosis factor (TNF-α), malondialdehyde (MDA) formation, myeloperoxidase (MPO) content, and number of infiltrating leukocytes in a MAPK/NF-κB–dependent manner in the lungs of septic animals challenged with *K. pneumoniae* (Tang et al., 2021). Similarly, AS101 (an inorganic compound with anti-apoptotic, anti-inflammatory, and immunomodulatory effects; Okun et al., 2007) has been described to reduce bacterial load across the liver, kidney, and spleen, and ultimately increasing mice survival from 0% 30 h after *K. pneumoniae* injection *i.p.* to 75% after 72 h in a mouse model of carbapenem-resistant *K. pneumoniae* sepsis (Yang et al., 2021). Also, TNF-related apoptosis-inducing ligand (TRAIL) encapsulated to a polypeptide-crosslinked nanogel has been described to significantly reduce bacterial load in blood and increase mice survival from 0% 4.5 days after *K. pneumoniae* instillation *i.t.* to 75% after 12 days in a mouse model of *K. pneumoniae* sepsis (Chen et al., 2019). Likewise, adipose-derived mesenchymal stem cells were described to significantly reduce bacterial load across the lung, blood, liver, and spleen; reduce pro-inflammatory cytokines (TNF-α, IL-1β, and IL-6) in the lung; and reduce tissue immune infiltration and damage, by downregulating genes related to nicotine, thymine, and uracil degradation, and upregulating genes related to unfolded protein response, sirtuin signaling pathway, and leukocyte adhesion and diapedesis, among others, in a mouse model of *K. pneumoniae* induced pneumosepsis (Perlee et al., 2019).

A cutting-edge approach using red blood cell membrane-coated poly(lactic-co-glycolic acid) (PLGA) nanoparticles (γ3-RBCNPs) to improve antibiotic bioavailability showed an astounding increase in mice survival from 0% 4 days after *K. pneumoniae* instillation *i.t.* to 60% after 7 days of instillation, with a significant and systematic reduction in bacterial load across the lung, blood, liver, spleen, and kidney, and a significant reduction in circulating levels of TNF-α, IL-1β, and IL-6 (Liu et al., 2022). Another cutting-edge approach using novel therapeutic antibodies targeting K1-serotype CPS of hypervirulent *K. pneumoniae* (hvKp) strains to promote bacterial phagocytosis by Kupffer cells in the liver were described to significantly reduce bacterial load across the lung, liver, and spleen, of hvKp challenged mice while increasing their survival from 0% 4 days after hvKp injection *i.p.* to 75% after 15 days in a mouse model of *K. pneumoniae* sepsis (Diago-Navarro et al., 2017). On the edge of knowledge, it has been revealed that exposure to blue light (peak 442 nm) significantly reduces bacterial load from the lung, blood, liver, and spleen, while significantly increasing mice survival from 15% 100 h after *K. pneumoniae* instillation *i.t.* to 62% after 125 h of bacterial challenge (Griepentrog et al., 2020). This was explained by local modulation of MPO activity and neutrophil abundance. Interestingly, there was no significant change in mononuclear abundance in the lung. The effect of blue light was mechanistically elucidated and attributed to a neural circuit signaling through a cholinergic anti-inflammatory pathway that directly controls immune responses in the spleen, enhancing control of the infection, and improving survival (Griepentrog et al., 2020). Thus, the potential therapeutic utility of something as trivial as ambient light in the ICU is strongly underscored. These cutting-edge approaches constitute powerful and promising tools in the search for new avenues for the treatment of sepsis that entails further research and encouragement. Lastly, pushing the edges of knowledge, it has been described that *K. pneumoniae* is able to induce alterations in the gut microbiome and cecal metabolome in a mouse model of sepsis (Wu T. et al., 2020). A significant difference in the richness, diversity, and composition of bacterial communities in mice challenged with *K. pneumoniae*, with fewer Bacteroidetes and Firmicutes but higher levels of Proteobacteria and Verrucomicrobia. At the genus level, mice challenged with *K. pneumoniae* had significantly fewer Bacteroides, Parabacteroides, Bifidobacterium, Clostridium, Coprococcus, and Prevotella, which produce short-chain fatty acids (SCFA). Interestingly, absolute concentration of SCFAs both in cecal contents and in serum were consistently lower in mice challenged with *K. pneumoniae*. Notably, supplementation of SCFAs in drinking water significantly reduced bacterial burden and tissue damage in the lungs, and significantly reduced mortality, while increasing phagocytic capacity of alveolar macrophages and pulmonary levels of IL-6 and TNF-α. Thus, characterization of microbial biomarkers displays a sizable potential for aiding in the diagnosis/prognosis of patients under critical care.

Additional evidence from animal models implicates PTX3 pentraxin (a component of humoral innate immunity involved in resistance to selected pathogens by promoting opsonophagocytosis) as protective factor expressed on the host side promoting bacterial phagocytosis, expression of TNF-α, IL-1β, IL-6, and CXCL-1, limiting MPO levels and leukocyte counts, protecting from tissue hemorrhage and increased mortality in septic mice challenged with *K. pneumoniae* (Asgari et al., 2021). Hypoxia-inducible factor (HIF) has also been implicated in modulating the immune system of the host during sepsis (Otto et al., 2021). Using a Cre/lox system, HIF1α was found instrumental to engage host defense against *K. pneumoniae* pneumosepsis for its significant role in bacterial load reduction both in the lung and distant organs and limiting levels of pro-inflammatory cytokines while enhancing the release of IL-10 in the lung (Otto et al., 2021). This modulation was explained by an energetic dysregulation affecting glucose uptake, leading to a significant reduction in TNF-α production in both alveolar and interstitial macrophages in LysM-cre × *Hif1α^fl/fl^* mice during onset *K. pneumoniae i.n.* challenge. Collectively, this frontline evidence shows as the foundation for the establishment of new diagnostic tools and the development of the therapeutics of the future.

## 4. Staphylococcus aureus

*Staphylococcus aureus* is a facultative Gram-positive organism mostly recognized for its pathogenic potential. Notwithstanding, *S. aureus* is a frequent colonizer in human hosts (Laux et al., 2019), with abundances reported recently as 32% for neonates (Arora et al., 2023), 25–29% for pediatrics (McNeil et al., 2022; Arora et al., 2023), and 30% for adults (Erayil et al., 2022). Although colonization is asymptomatic, it has been deemed a risk factor for developing BSI in the event of pathogenic transformation and bloodstream invasion (Tacconelli et al., 2017). Currently, there is no consensus on what single event leads to pathogenic progression. Rather, virulence mechanisms appear to be related to circumstance, environmental opportunity, and particularly, to host-pathogen interplay (Kwiecinski and Horswill, 2020).

### 4.1. Epidemiology

**Figure 1:**
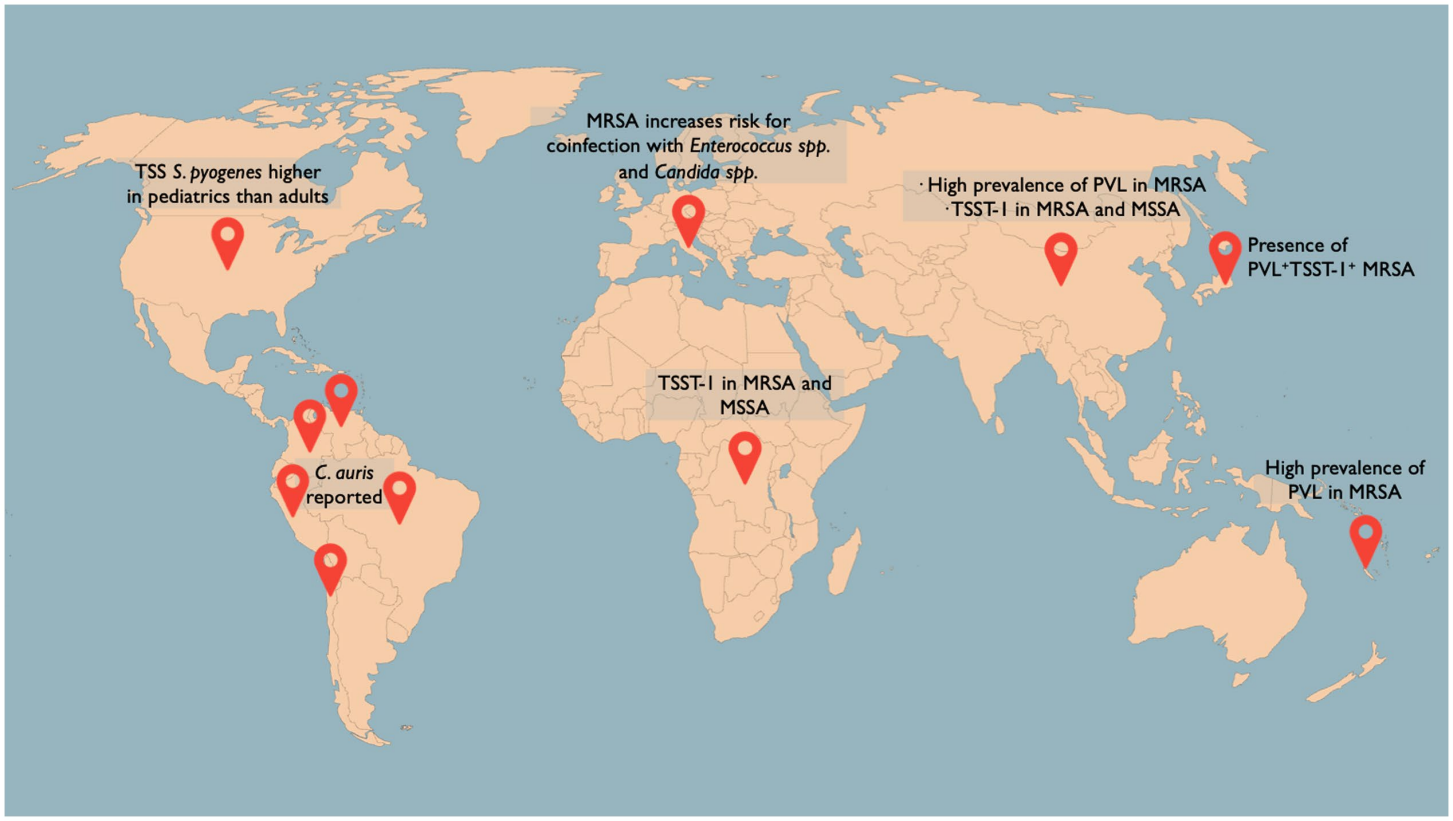
Selection of the latest figures on sepsis-associated pathogen epidemiology worldwide. *Streptococcus pyogenes*-associated TSS is reported in the United States with an uneven distribution between adults (22.5%) and pediatrics (7.1%; Meehan et al., 2018). TSST-1 has been identified in 67% of MRSA and 4% of MSSA blood isolates in the Democratic Republic of the Congo (Vandendriessche et al., 2017) and in 23.1% of MRSA and 6.7% of MSSA blood isolates in China (Liu et al., 2018). PVL ^+^ TSST-1^+^ isolates have been found in 0.4% of all MRSA isolates in Japan (Kaneko et al., 2023). A high prevalence of PVL^+^ MRSA strains in adults has been found in New Caledonia Island (Imauven et al., 2022). An escalating number of countries in Latin America have reported the detection of *Candida auris* (Riera et al., 2022).

*Staphylococcus aureus* is reported as the leading causative agent for BSI in North America and the second most prevalent for the rest of the world (Diekema et al., 2019). Moreover, a recent study conducted in Australia reported an increased incidence of *S. aureus* bacteremia in older men, especially over 60 years old, almost doubling the incidence reported in the same age group in women (Imam et al., 2019). Furthermore, another study reported that MRSA bacteremia in patients older than 75 years old represents a significant risk factor (OR = 2.4) for 30-day mortality (Bassetti et al., 2018). Similarly, a prospective observational study carried out in Australia and New Zealand found a strong majority of the samples associated to advanced age (>70 years-old, 63.1%), male sex (64%), and methicillin susceptibility (75.9%), and multivariate analysis further revealed sepsis as the most influent risk factor for 30-day mortality in patients with *S. aureus* bacteremia (OR = 4.01; Turnidge et al., 2009). Bloodstream coinfection with other pathogenic agents is less frequent, although it has been reported that *S. aureus*/*Candida albicans* coinfection has a catastrophic proportion of 82% for 30-day mortality and a high correlation with indwelling vascular devices (Wu Y. M. et al., 2021). In fact, a retrospective observational study conducted in Italy showed that patients older than 80 years afflicted with MRSA BSI had the highest risk for 30-day mortality and coinfection with *Enterococcus* spp. and *Candida* spp. were significant risk factors for this outcome (Giovannenze et al., 2021; Figure 1). More recently, *S. aureus*/SARS-CoV-2 coinfection has gained more insight, with reported 30-day mortality of 67%, largely attributable to hospital-onset bacteremia in patients mostly under mechanical ventilation (Cusumano et al., 2020).

### 4.2. Pathogen factors

A recent study comparing the pathogenic mechanisms of fixed clonal complexes of *S. aureus* during sepsis showed only one virulence property that remained consistent (Recker et al., 2017). These were polymorphisms in the *capA* gene, which encodes an enzyme involved in capsule biosynthesis, which is responsible for host immunity protection and acts as a virulence factor during sepsis. Notably, this gene is absent in *S. aureus* clones in North America (Boyle-Vavra et al., 2015), further highlighting the absence of a consistent set of virulence factors and the overall versatility of this pathogen. Another virulence factor of interest is cytotoxin Panton–Valentine Leukocidin (PVL), which is highly prevalent in community-acquired methicillin-resistant *S. aureus* (MRSA) and has been associated with higher risk of developing sepsis (Ahmad et al., 2020; Monecke et al., 2020). A study conducted in China comparing MRSA strains isolated from adult and pediatric patients reported a higher prevalence of PVL in adult MRSA isolates (55.8 vs. 35.3%), in addition to higher antibiotic resistance and higher mortality in adults (Zhao et al., 2022; Table 1). Clone ST5/ST764-MRSA SCC*mec* II was the predominant isolate in adults, whereas clone ST59-MRSA SCC*mec* IV was the predominant isolate in pediatrics (Zhao et al., 2022). Also regarding PVL, another study comparing adult and pediatric isolates from patients admitted to the ICU in New Caledonia Island (Southwest Pacific region) reported a significantly higher prevalence of PVL^+^ MRSA strains in adults (61 vs. 30%), which carried an implicit higher risk for developing sepsis and a fatal outcome (OR = 4.57; Imauven et al., 2022).

Added to the array of virulence factors of *S. aureus* is toxic shock syndrome toxin-1 (TSST-1), which relates to the rapid development of symptoms consistent with sepsis, including hypotension and organ dysfunction. Although there is an insufficient number of large-scale studies on TSST-1 molecular epidemiology, recent data from the Democratic Republic of the Congo identified TSST-1 in 67% of MRSA and 4% of MSSA blood isolates (Vandendriessche et al., 2017), while data from China reports TSST-1 in 23.1% of MRSA and 6.7% of MSSA blood isolates (Liu et al., 2018). For their joint virulence and its inherent potential to cause a fatal outcome (Hayakawa et al., 2020), despite representing only 0.4% of all MRSA isolates in a whole-genome analysis carried out in Japan (Kaneko et al., 2023) and 0% of all isolates in a worldwide comparative genomic analysis (Zhou et al., 2021), systematic monitoring of PVL^+^TSST-1^+^ isolates becomes a relevant facet of *S. aureus* virulence to survey. A summary of the factors detailed in this section is listed in Table 2.

### 4.3. Host factors

Host immunity plays a pivotal role in clearing *S. aureus* presence in the bloodstream. Notably, neutrophils isolated from patients undergoing *S. aureus* sepsis exhibit increased formation and release of neutrophil extracellular traps (NET) and pro-inflammatory cytokines TNF-α, IL-1β, and IL-8 (Gupta et al., 2022). Moreover, neutrophil and lymphocyte abundance as a ratio has been demonstrated as an independent predictor for 90-day mortality. Such simple but relatively costly measurement shows potential as a prognostic tool, which if supplemented with appropriate sensitivity and specificity information, may prove useful in orienting the management of septic patients (Greenberg et al., 2018). Likewise, serum levels of C5a and IL-10 were found significantly higher in samples from *S. aureus* bacteremic patients when compared to matched hospitalized and community controls (Eichenberger et al., 2020). Also, in the quest for prognostic predictors during *S. aureus* bacteremia, activity of caspase-1 in neutrophils and monocytes, serum levels of IL-18, and whole blood mRNA levels of inflammasome mediator NOD-like receptor family pyrin domain-containing 3 (NLRP3), were found to display a significant difference between survivors and non-survivors (Rasmussen et al., 2019). Bioinformatic screening of blood samples of *S. aureus* bacteremic patients further reveals direct correlation between the expression of a set of five genes (*COX7C, NDUFA4, ATP5J, NDUFB3*, and *COX7A2*; relating to aerobic respiration, cellular stress response, mitochondrial electron transport, mitochondrial transport, and oxidative phosphorylation) and adverse outcome (Wu H. et al., 2021). Although further testing of the prognostic value of such analysis is required, the high-throughput approach is noteworthy and the potential of a multiplex approach to follow the development of sepsis is promising.

Similarly, meta-analysis of existing databases further corroborates the plausibility for bioinformatics approaches to aid in tackling sepsis from a molecular flank. Analysis of post-translational modifications to mRNA (in particular, methylation of the sixth N atom on the adenine base, m6A) integrated into single nucleotide polymorphisms (SNP) revealed a significant presence of a set of m6A-SNPs during *S. aureus* sepsis (Sun et al., 2020; Table 1). Such set was characterized and associated to DNA repair, vesicle-mediated transport, peptidyl-serine phosphorylation, leukocyte migration, catabolic processes, regulation of endopeptidase activities, phagocytosis, and platelet degranulation. Although no further clinical characterization was reported, the value in diagnostics, prognosis, and therapeutics is yet to be explored.

Host defense against *S. aureus* is not limited to leukocytes, as recently demonstrated in a translational report (Sun et al., 2021). Prompted by an observation of the strong association between thrombocytopenia (without leukopenia or leukocytosis) and patient mortality, authors used an *in vivo* model to demonstrate that the underlying mechanism of platelet-mediated antibacterial activity is impaired by *S. aureus* pore-forming α-toxin (*Hla*). Interaction of α-toxin with platelet chemoreceptor P2Y12 was inhibited by using FDA-approved and commercially available antiplatelet drug ticagrelor, resulting in extended protection from thrombocytopenia, enhanced bacterial clearing, protection from organ damage, and increased survival. Thus, in a single report platelet contribution to host immunity and the repurpose of an available drug to protect against *S. aureus* bacteremia was elegantly demonstrated. Not surprisingly, ulterior reports have extended these findings by corroborating the protective effect of ticagrelor against *S. aureus* bacteremia. In a nationwide observational cohort study carried out in Denmark, ticagrelor use was demonstrated protective not only against *S. aureus* bacteremia but also, in a higher proportion, against sepsis and pneumonia (Butt et al., 2020). Further evidence on ticagrelor repurposed use is described in a recent case report detailing a steep correction in platelet count and complete recovery of a patient afflicted with methicillin-sensitive *S. aureus* (MSSA) bacteremia treated with ticagrelor for 3 months (Ulloa et al., 2021). A summary of the factors detailed in this section is listed in Table 3.

### 4.4. Treatment

Empiric treatment for *S. aureus* is initiated pending susceptibility tests and is prophylactically directed against MRSA with the use of vancomycin or daptomycin (Liu et al., 2011). Evidence has been presented in favor of the use of initial doses of vancomycin ≥20 mg/kg for a faster resolution of systemic inflammation (Wesolek et al., 2018). Once susceptibility is elucidated, if isolate is MSSA, antibiotic treatment is de-escalated to a β-lactam agent. Use of combination therapy is controversial, with weak evidence found in a recent clinical trial (Pujol et al., 2020) and several cohort studies (Rieg et al., 2017; Davis et al., 2018; Guthridge et al., 2021; Kufel et al., 2023). Appraisal of the therapeutic value of monotherapy was presented in a recent clinical trial carried out in South Korea, in which treatment of MSSA bacteremia with nafcillin was associated with higher Sequential Organ Failure Assessment (SOFA) scores, higher rates of treatment failure, and astoundingly higher mortality figures than treatment with cefazolin (Lee et al., 2018). In fact, odds ratios for treatment with cefazolin over nafcillin were 0.39 for SOFA score ≥ 2, 0.43 for treatment failure, and 0.15 for 90-day mortality. Overall protective effect of treatment of MSSA bacteremia using cefazolin was quantified with an adjusted OR = 0.44. Interestingly, matched propensity scores resulted significant only for the bacteremic group and not for the septic group, highlighting the higher level of complexity in the pathobiology of sepsis. A relevant preoccupation is the increasing resistance to methicillin, where median percentage resistance increased from 16.6% in 2017 to 18.3% in 2020, according to a recent global report (World Health Organization (WHO), 2022a). Other reports on antimicrobial resistance related to sepsis describe *S. aureus* methicillin resistance to be 17.6% in Australia and 15.5% in Europe (Coombs et al., 2022). Management of MRSA by means of either monotherapy or combination therapy of vancomycin or daptomycin, with or without a β-lactam agent, shows no significant differences in 30-day mortality risk, length of stay, or risk of persistence, as evidenced by a recent meta-analysis (Yi et al., 2021). A fact to be assessed in terms of harms versus benefits when sizing therapeutical value and antibiotic resistance.

## 5. Pseudomonas aeruginosa

*Pseudomonas aeruginosa* is an aerobic Gram-negative bacterium distributed ubiquitously in the environment. *P. aeruginosa* infections are frequently nosocomial and opportunistic, as they frequently occur concomitantly to existing physical, phagocytic, or immunologic dysfunctions in host defense, leading to bloodstream, urinary tract, respiratory, and intra-abdominal infection (Tang et al., 2017; Li X. et al., 2023).

### 5.1. Epidemiology

Recent reports place *P. aeruginosa* as the fourth most common cause of BSI globally (Diekema et al., 2019) and the third most frequent pathogen isolated in catheter-associated urinary tract infection and ventilator-associated pneumonia (Weiner-Lastinger et al., 2020). This is relatively consistent throughout the world, except in North America, where *P. aeruginosa* BSI ranks fifth (Diekema et al., 2019). Despite its relatively low proportion in BSI incidence, a recent study analyzing isolates from the United States reports *P. aeruginosa* as the second most frequent pathogen causing BSI mortality, accounting for nearly a quarter of the cases between 2016 and 2020 (Ohnuma et al., 2023).

### 5.2. Pathogen factors

Although pathogenesis of *P. aeruginosa* has been extensively studied in models of pneumonia, burn wounds and urinary infection, few studies have explored virulence factors expressing in sepsis-inducing strains. A recent transcriptomic analysis of clinical isolates associated to BSI revealed high-level expression of a novel cell-surface signaling system named Hxu (a response system for sensing extracellular porphyrin rings of haem; Otero-Asman et al., 2019), whereas deletion or overexpression of this pathway resulted in reduced or enhanced BSI, respectively (Yang F. et al., 2022). Several genes related to *P. aeruginosa* metabolism have been associated with the level of virulence of the strain PAO1, extensively used in sepsis models. Deletion of the regulator of α-ketoglutarate transport *mifR* resulted in a significant improvement of survival following pneumonia-induced sepsis in a murine model as well as a reduction in pro-inflammatory cytokines and reduced NLRP3 inflammasome activation (Xiong et al., 2022). In addition, expression of enzymes able to degrade host extracellular matrix components such as heparinase contribute to the virulence of sepsis-inducing strain *P. aeruginosa* PA14. Mutation of the gene encoding for heparinase (*hepP*) impaired bacterial dissemination and prevented mortality in murine models of thermal injury and intraperitoneal PA14 injection, indicating that *hepP* contributes to the pathogenesis of PA14 (Dzvova et al., 2018). A summary of the factors detailed in this section is listed in Table 2.

### 5.3. Host factors

Recent studies indicate that susceptibility to develop systemic infection is associated with various predisposing host factors. In fact, immune compromised hosts exhibit an adjusted odds ratio of 3.7 for BSI in a multivariate logistic regression model considering age, sex, ethnicity, chronic comorbidities, source of infection, recent ambulatory procedures, urinary catheterization, residence in skilled nursing facilities, chronic hemodialysis, current and recent hospitalization, and prior exposure to β-lactams and fluoroquinolones in the past 90 days (Hammer et al., 2017). Moreover, the latter odds ratio has been reported as high as 13.82 for BSI and 23.1 for 30-day mortality, also in multivariate analyses (Tan et al., 2021). Interestingly, according to a 2019 report on community-onset *P. aeruginosa* urinary infection in elderly people, odds ratio for sepsis were highest for patients who received healthcare (OR = 5.52), who had an indwelling urinary catheter (OR = 3.25), and who were male (OR = 3.16), as determined in a stepwise logistic regression (Esparcia et al., 2019). An interesting instance of host susceptibility is presented by the growing number of cases reported on *P. aeruginosa* sepsis in pediatric hosts with X-linked agammaglobulinemia (XLA, OMIM #300755; Bhardwaj et al., 2017; Biscaye et al., 2017; Birlutiu et al., 2019; Huang H. et al., 2020), an immunodeficiency characterized by failure to produce mature B lymphocytes resulting in clinically undetectable levels of all immunoglobulin isotypes. Pediatric patients with XLA rapidly develop sepsis after *P. aeruginosa* infection and, while specific pathogen identification delays targeted therapy, the appearance of landmark skin lesions (ecthyma gangrenosum) represent an informative sign of the underlying causative agent (*P. aeruginosa*).

An additional useful example that warrants attention is the protective effect observed for hemoglobin levels in *P. aeruginosa* pediatric sepsis as an outcome, with an astounding OR = 0.155 obtained after multivariate logistic regression analyses in a matched case–control study conducted in Taiwan (Kung et al., 2020). Extensive research has characterized the immune responses at the lung and skin in mouse models of acute *P. aeruginosa* infection but the role of both innate and adaptive immune cells during *P. aeruginosa* sepsis is still poorly understood. Bacterial dissemination and sepsis are usually accompanied by increased numbers of phagocytes, but in a model of burn wound infection, neutrophil and monocyte recruitment to the seroma fail to contain *P. aeruginosa* dissemination, promoting the development of sepsis (Brammer et al., 2021). However, modulation of these cells by host-derived molecules may prevent severe sepsis and mortality. Endogenous hydrogen sulfide (H_2_S) produced by cystathionine-γ-lyase enhances neutrophil recruitment and their phagocytic activity resulting in reduced mortality in a mouse model of sepsis induced by a multidrug-resistant strain. In addition, H_2_S reduces the expression of *P. aeruginosa* quorum sensing genes, favoring pathogen phagocytosis. Moreover, clinical correlates show that patients who survived sepsis had higher levels of circulating H_2_S compared to non-survivors, strongly suggesting that H_2_S plays a protective role during *P. aeruginosa* sepsis (Renieris et al., 2021).

Host immunosuppressive mechanisms may also play a detrimental role during *P. aeruginosa* sepsis. Animal models of secondary bacteremia following cecal punction ligation exhibit improved survival after a partial deletion of Tregs and a reduction in anti-inflammatory cytokine IL-10 (Hu et al., 2018). In line with this, treatment with the immunoregulatory molecule ethyl pyruvate reduced lung levels of IL-10 and the expression of *FOXP3* in lung-derived Tregs in a two-hit model of sepsis, reversing *P. aeruginosa* secondary pneumonia (Chen et al., 2017). IL-10 deficient mice are more susceptible to PA14-induced pneumonia but do not display bacteremia (Belo et al., 2021), suggesting that IL-10 helps to control local antimicrobial responses, but high lung levels may favor bacterial dissemination. Therefore, the role of IL-10 and other anti-inflammatory host-derived molecules during *P. aeruginosa* sepsis is poorly understood and requires further research to confirm its role increasing host susceptibility to severe sepsis and mortality. *P. aeruginosa* not only interacts and promotes colonization and coinfection with *S. aureus* (Clancy et al., 2014) and *Stenotrophomonas maltophilia* (McDaniel et al., 2020) but is also a common cause of secondary bacterial infections in patients hospitalized for COVID-19 (Lansbury et al., 2020; Garcia-Vidal et al., 2021), a fact exemplifying its opportunistic nature. *P. aeruginosa* is associated with high in-hospital mortality rates and prolonged lengths of stay (Naylor et al., 2018). A summary of the factors detailed in this section is listed in Table 3.

### 5.4. Treatment

Clinically, antimicrobial treatment proceeds observing four arms: controlling the source (i.e., infected catheters or ventilator reservoirs), timely initiation of therapy, use of mono/combination therapy, and limiting antibiotic resistance. Extensive and uncontrolled use of antibiotics has contributed to the increase in multi-drug resistant (MDR) and extensively-drug resistant (XDR) strains, which are difficult to treat, especially in cases of severe sepsis. In fact, *P. aeruginosa* is the fourth leading pathogen behind all deaths attributable to antibiotic resistance in high-income countries (Murray et al., 2022). Genetic characterization of *P. aeruginosa* bacteremia at advanced ages indicates a significant presence and association with fatal outcome of resistance genes *bla*_GES_, *aadB*, *gyrA* (T83I), *parC* (S87L), virulence gene *exoS*, multilocus sequence typing (MLST) ST235, O-antigen serotype O11 (Recio et al., 2021), strong biofilm producing (di Domenico et al., 2021), and non-motile strains (Gupte et al., 2021). Interestingly, recent prospective study indicates that carbapenem resistance is not significantly related to treatment failure, a fact likely related to the site of infection, host susceptibility, and clinical severity (Lee C. M. et al., 2022). Recent studies in patients have shown inconclusive about the benefit of the combination of antibiotics such as ceftazidime-avibactam against MDR and XDR strains (Corbella et al., 2022). Although ceftolozane/tazobactam is effective against complicated urinary tract infections and complicated intra-abdominal infections, univariate analyses reveal that treatment using this therapeutical combination is significantly associated with a successful management of the clinical presentation of sepsis (Bassetti et al., 2019). Additionally, early use of this combination in neutropenic patients has been shown to control progression of skin infection, suggesting a protective effect on further dissemination with or without sepsis (Coppola et al., 2020).

By translation, a recent *in vivo* study explored the efficacy of different antibiotics in mouse models of sepsis caused by *P. aeruginosa*. Although carbapenem combinations did not show improved efficacy against carbapenemase-producing *P. aeruginosa*, meropenem monotherapy showed promising *in vivo* efficacy against peritoneal sepsis (Herrera-Espejo et al., 2022). Interesting evidence from *in vivo* models reveals that peptidylarginine deiminase (PAD) type 2 deficiency (*Pad2^−/−^*) significantly improves survival in *P. aeruginosa* pneumonia-induced sepsis by attenuating acute lung injury (Wu Z. et al., 2020). Under the proposed mechanism, PAD2 deficiency enhances bacterial clearance by reducing caspase-1-dependent pyroptosis in bone marrow-derived macrophages. Thus, PAD2 is revealed as a promising molecular target that warrants further research.

Other experimental therapies have been tested alone or in combination with antibiotics in murine models and clinical assays. Because *P. aeruginosa* binds to host epithelial cells via pili and type-IV pili (T4P) are crucial for bacterial twitching, attachment, biofilm formation, and motility (particularly T4P containing PilA subunits; Zahedi bialvaei et al., 2021a), mAb against disulfide turn region of PilA (QA) and PilQ, alongside with clinically available antibiotics levofloxacin, ceftazidime, and gentamicin, showed a synergistic effect in the treatment of a mouse model of *P. aeruginosa* sepsis by preventing bacterial dissemination and increasing overall survival (Zahedi bialvaei et al., 2021b). Similarly, a case describing the use of a combination of antibiotics and a cocktail of two phages (PNM and 14–1) to attend an infection with an XDR strain secondary to liver transplantation in a septic toddler was deemed successful after confirming full clearance of *P. aeruginosa* from the bloodstream (van Nieuwenhuyse et al., 2022). Moreover, *in vitro* studies using the phage PNM have shown a synergistic effect in combination with suboptimal concentrations of colistin, aztreonam, or gentamycin against clinical isolates of *P. aeruginosa* (van Nieuwenhuyse et al., 2022).

## 6. Streptococcus pyogenes

*Streptococcus pyogenes* is a facultative anaerobe Gram-positive bacterium that strictly infects humans (Gera and McIver, 2013). *Streptococcus pyogenes* infections are highly contagious and range from pharyngitis, and skin or soft tissue (non-necrotizing) infection, to respiratory tract infection, pregnancy-associated infection, necrotizing fasciitis, and bacteremia with toxic shock syndrome (TSS; Walker et al., 2014).

### 6.1. Epidemiology

Recent analyses report an increasing incidence of *S. pyogenes* infections with a high case fatality in the older population (Shakoor et al., 2017; Meehan et al., 2018; Blagden et al., 2020; Vilhonen et al., 2020; Bläckberg et al., 2022; Thomson et al., 2022). The most prevalent form of clinical presentation of invasive *S. pyogenes* infection is bacteremia (75%), followed by focus without bacteremia (19%) and necrotizing fasciitis (7%; Meehan et al., 2018). Moreover, TSS is reported in 19% of bacteremia cases with an uneven distribution between adults (22.5%) and pediatrics (7.1%; Meehan et al., 2018), the latter figure reported higher in the United States (16.9%; Gaensbauer et al., 2018). Nevertheless, *S. pyogenes* bacteremia is significantly less represented (<8%) when compared to other pathogens (Ferreira et al., 2023; Tabah et al., 2023).

### 6.2. Pathogen factors

Until recently, the best described virulence factor of invasive *S. pyogenes* infection is the M protein, encoded by the *emm* gene and expressed on the bacterial surface. A recent Spanish report on *emm* diversity in pediatrics revealed clone *emm1*/ST28 as the most prevalent and the most consistently detected over the 12 years span of the report, followed by clones *emm12*/ST36-ST242 and *emm6*/ST382 (Sánchez-Encinales et al., 2019). This trend was echoed by findings of a retrospective cohort study in Sweden, which included 286 samples from adult patients collected in a 4-year period (Bläckberg et al., 2022) and a prospective German nationwide cohort study, which included 719 isolates from patients of all ages collected in a 6-year period (Imöhl et al., 2017). A different pattern was observed in Finland where, in a recent cohort study spanning 12 years and focusing on women of childbearing age, *emm28* showed as the most frequent type of *S. pyogenes* strain, displaying a significant association with delivery and puerperium-related infections leading to bacteremia (Gröndahl-Yli-Hannuksela et al., 2021). Other proposed virulence factors arising during invasive *S. pyogenes* disease include streptococcal superantigens *speG*, *speH*, *speJ*, and *speK*, which, in the presence of certain underlying comorbidities (i.e., diabetes, chronic skin lesions, liver dysfunction, and respiratory distress), modulate the risk of invasive *S. pyogenes* disease (Imöhl et al., 2017).

Evidence from *in vivo* studies highlight the relevance of non-canonical Tyr-phosphatase M5005_Spy_1476 as a molecular mediator for *S. pyogenes* pathogenesis, an enzyme reported to maintain this pathogen in a virulent state leading to increased subject mortality by modulating its ability for adherence and invasion of host cells, and for *in vitro* biofilm formation in a mouse model of sepsis (Kant and Pancholi, 2021). Similar evidence has been reported for predicted gene *spy1343*, which codes for an inferred 298-amino acid protein that belongs to the LysR family of DNA-binding transcriptional regulators. Interestingly, mice challenged with a mutant strain carrying a deletion of *spy1343* exhibited a significant increase in mortality when compared to their control (WT) counterparts (Sitkiewicz and Musser, 2017). Explanation for this excess of virulence lies in the control of *spy1343* over genes that participate in short-chain fatty acid metabolism, which have been linked to overall bacterial pathogenesis and overall virulence. Additional evidence from *in vivo* studies reveals that *S. pyogenes* virulence may be traced from a clinical standpoint by evaluating the pattern of leukocyte and platelet abundance. Multidimensional scaling of median values of absolute counts of such type of cells was able to discriminate clusters of virulence and disease progression over time with precision, using simple, routinary readouts in experimental infection using a small animal model. Thus, underscoring the potential and unprecedented diagnostic/prognostic value of computer science in human infection progression, particularly in sepsis (Loof et al., 2018; Table 1). A summary of the factors detailed in this section is listed in Table 2.

### 6.3. Host factors

Host immune responses during *S. pyogenes* sepsis has received modest attention in both *in vivo* and *in vitro* models. It has been proposed that vascular endothelial growth factor (VEGF) expressed in endothelial cells may promote antimicrobial response against *S. pyogenes* infection, as observed *in vitro* with human endothelial cells increasing lysosomal biogenesis and function and overall bacterial xenophagy against *S. pyogenes* through the activation of TFEB and its downstream genes (e.g., *ATPV6* and *LAMP1*), and *in vivo* with VEGF treatment significantly increasing subject survival rate (Lu et al., 2022). Moreover, patients with severe invasive disease (sepsis, bacteremia, necrotizing fasciitis, and TSS) exhibit significantly lower serum levels of VEGF compared to those with non-invasive disease (Lu et al., 2022). Also relating to host immune response, TLR signaling plays an important role in early immune reactions against *S. pyogenes*. Nucleic acid detection by the endosomal receptor TLR13 mediates immune cell activation, expression of pro-inflammatory cytokines, and formation of ROS and reactive nitrogen species (RNS) against *S. pyogenes in vitro*. Mice defective in endosomal signaling (*Unc93b1^−/−^*) exhibited higher bacterial burden at sites of lesion and the spleen, and significantly increased systemic inflammation in a murine model of soft tissue infection. Therefore, endosomal TLR signaling may play an important role in activating antimicrobial responses at local infection sites and prevent systemic disease (Hafner et al., 2019). Several studies have reported a synergistic interaction for infection between *S. pyogenes* and pathogenic viruses of the respiratory tract (Brundage, 2006; Herrera et al., 2016), a fact made evident during the 1918 influenza pandemic (Morens and Fauci, 2007), the 2009 H1N1 influenza pandemic (Jean et al., 2010), and more recently, the 2019 SARS-CoV-2 pandemic (Khaddour et al., 2020). However, data on *S. pyogenes* superinfections are scarce and interactions with additional infective agents warrant further resource allocation and research (Turner, 2022). A summary of the factors detailed in this section is listed in Table 3.

### 6.4. Treatment

Empiric antimicrobial therapy typically initiates pending culture results to then be tailored accordingly. Penicillin monotherapy in the setting of high inoculum has been associated with treatment failure (Stevens et al., 1994). For this reason, adjunctive use of clindamycin is recommended for its strong association with lower mortality (OR = 0.44; Babiker et al., 2021). An increasing number of isolates with resistance to clindamycin and other macrolides have been consistently identified around the globe [e.g., the United States (DeMuri et al., 2017), Hungary (Gajdács et al., 2020), India (Jayakumar et al., 2022), and China (Lu et al., 2017)] with the surprising exception of Spain, where tetracycline, erythromycin, and clindamycin resistance rates declined between 2007 and 2020 (Villalón et al., 2023).

Owing to its incapacity for horizontal gene transfer, *S. pyogenes* resistance to β-lactam antibiotics is a feature considered rare (Hayes et al., 2020). However, a report by the Active Bacterial Core surveillance (US Centers for Disease Control and Prevention, CDC) has recently made the alarming discovery of a *S. pyogenes* strain (*emm43.4*/PBP2x-T553K) with increased β-lactam resistance (Chochua et al., 2022; Table 1). Similarly, a recent report from active surveillance in Israel described the emergence of an outbreak of a new MDR strain *emm*93.0 responsible for an unusually large number of invasive *S. pyogenes* infections, especially present in the bloodstream (Ron et al., 2022). These unprecedented findings underline the importance of population-based pathogen surveillance programs internationally. Nonetheless, there is consensus that *S. pyogenes* infections—even when invasive—are associated with a low attributable mortality, unless they are invasive and meet the criteria for TSS, for which cases the mortality rate can reach up to 44% (Schmitz et al., 2018).

## 7. *Candida* species sepsis

Invasive fungal infections are clinical manifestations of fungal infections different from superficial infections proven by culture and isolation from sterile sites such as deep tissue, cerebrospinal fluid, or blood (Donnelly et al., 2019).

### 7.1. Epidemiology

*Candida* species are the predominant cause of life-threatening invasive fungal infections in hosts with decreased defenses (e.g., immunocompromised individuals, patients who have endured invasive clinical procedures, or have experienced major trauma; Bongomin et al., 2017) and constitute the most common cause of fungal BSI (Pappas et al., 2016), a clinical condition that has experienced a significant and concerning increase over the last decade, as a retrospective cohort study comprising 465 candidemia episodes in Germany (Mohr et al., 2020) and a retrospective cohort study comprising 170 candidemia episodes in Switzerland (Battistolo et al., 2021) report. Although the latest report by the SENTRY Antifungal Surveillance Program carefully details different forms of invasive fungal infection, it does not present isolated data for fungal BSI (Pfaller et al., 2019). Nevertheless, recent observational data ratify *Candida albicans* as the leading species during fungal BSI and further expand the list with other non-albicans species [in hierarchical order: *Candida parapsilosis*, *Candida glabrata*, and *Candida tropicalis* (Doğan et al., 2020); *Candida tropicalis*, *Candida parapsilosis*, and *Candida glabrata* (Al-Musawi et al., 2021)]. However, in one study from Saudi Arabia (Aldardeer et al., 2020) and another from India (Lamba et al., 2021), *Candida albicans* was relegated to the second place, being *Candida glabrata* and *Candida tropicalis* the leading species for fungal BSI, respectively.

### 7.2. Pathogen factors

*Candida* species grow in yeast or filamentous (pseudohyphae and hyphae) morphologies and this morphology strongly correlates to its pathogenicity, revealing fungal programs of invasion, virulence, and overall versatility and capacity for adaptation (Lo et al., 1997; Bartie et al., 2004). For a formidable review on pathogen virulence factors and the immune response during *Candida* sepsis we strongly recommend consulting article by Patricio et al. (2019). One such factor is (1,3)-β-D-Glucan (BDG), a major structural component of the inner cell wall in *Candida* species, which not only serves as the main pathogen-associated molecular pattern that interacts with pattern recognition receptors on the host side but has also been implicated a prognostic value. Although without accuracy or performance test for comparison with other diagnostic methods, BDG^−^ candidemia has been recently identified as an independent protective factor from poor clinical outcome (Agnelli et al., 2019).

### 7.3. Host factors

Correspondingly, timely removal of central venous catheter has also been identified as an independent protective factor against mortality (Kutlu et al., 2022). Incidentally, delayed removal of central venous catheter during candidemia has been consistently identified as a risk factor for mortality in adults (Huang H. Y. et al., 2020; Lee et al., 2020), pediatrics (Lee W. J. et al., 2022), and neonates (Chen Y. N. et al., 2022). Other risk factors for adult mortality with great representation across quantitative multivariate reports are concomitant bacteremia (Lee et al., 2020; Pieralli et al., 2021; Zhong et al., 2022; Gebremicael et al., 2023), high SOFA score (Bienvenu et al., 2020; Huang H. Y. et al., 2020; Jung et al., 2020; Kutlu et al., 2022), presence of liver cirrhosis (González-Lara et al., 2017; Bartoletti et al., 2021; Battistolo et al., 2021; Meyahnwi et al., 2022), kidney dysfunction (Poissy et al., 2020; Mazzanti et al., 2021; Kutlu et al., 2022), and others summarized in Table 3 (Bassetti et al., 2020; Yoo et al., 2020; Kim et al., 2021). Particularly for early-age groups, additional risk factors for mortality include breakthrough candidemia (Lee W. J. et al., 2022), previous use of antibiotics for >2 weeks, persistent candidemia, and preterm gestation (<32 weeks; Eisi et al., 2022), placing the emphasis in the implementation and follow-up of protocols to properly manage neonatal care.

Chronic comorbidities appraised as compounded scores (i.e., Karnofsky Performance Status <70 and Charlson Comorbidity Index ≥4) were also found to increase the risk of mortality during candidemia (Bassetti et al., 2020; Yoo et al., 2020; Kim et al., 2021; Vázquez-Olvera et al., 2023). Mixed *Candida*/bacterial BSI is referenced to occur in 18–56% of candidemia cases (Bouza et al., 2013; Kim et al., 2013; Chen et al., 2020) and recent articles agree [20.5% of candidemias reported in (Zhong et al., 2020) and 29.7% of candidemias reported in (Lee E. H. et al., 2022)]. The most frequent bacteria isolated from the bloodstream were coagulase-negative *Staphylococcus*, followed by *Klebsiella pneumoniae*, and *Staphylococcus aureus*. As expected, mixed BSI poses an even more complex challenge to host responses and, although a significant increase in mortality would be expected, the only significant increments were seen in length of ICU stay and length of mechanical ventilation use (Zhong et al., 2020). Conversely, *Candida* BSI following admission and treatment for COVID-19 has been found to significantly shift the outcome of patients toward fatality, along increasing the utilization of mechanical ventilation, the need for central venous catheter and parenteral nutrition, and overall length of stay (Rajni et al., 2021; Kayaaslan et al., 2023). Interestingly, admission and treatment for COVID-19 was found to double the incidence rate of developing candidemia, as a retrospective cohort study report (Kayaaslan et al., 2021). Even more interestingly, candidemia was found in a significant portion of patients who underwent surgery during their treatment for COVID-19 and those with a history of corticosteroid use. In fact, prior corticosteroid use was identified as a significant risk factor for mortality with a strength of association (OR = 4.4) comparable to advanced age (≥65 years old, OR = 5.6) and presence of sepsis (OR = 7.6), as demonstrated by multivariate analyses. The detrimental effect of prior corticosteroid use is a novel finding that requires further research for its potential value as a prognostic tool at COVID-19 admission. Other predictors of mortality for COVID-19 patients with candidemia identified through multivariate analyses include increased length of stay, high levels of D-dimer, use of tocilizumab (Rajni et al., 2021), ECMO support (Alessandri et al., 2023), high SOFA score (Omrani et al., 2021), and presence of a central venous catheter (OR = 19.07; Kayaaslan et al., 2023).

Several approaches for prediction and prognosis have proposed different combinations of readouts to match and even surpass current prognostic tools. Of note, accordingly to the era of data science, a machine learning-based algorithm using a myriad of clinical variables as input was able to predict candidemia with an AUC of 87.4% (Ripoli et al., 2020), an extraordinary magnitude that outperforms not only classical stepwise multivariable logistic regressions performed in the same report but also other independent studies proposing different arrangement of variables, i.e., the delta neutrophil index (with an AUC of 80.4%; Park et al., 2020) and a multivariate conditional regression-based risk score (with an AUC of 76.8%; Poissy et al., 2020). Mortality has also been appraised as an outcome, with a composite score (consisting of a combination of the SOFA score and the Charlson Comorbidity Index) reported to outperform (AUC of 79%) the independent performance of its components (AUC of 77% for SOFA score, AUC of 69.7% for Charlson Comorbidity Index; Asai et al., 2021; Table 1). A summary of the factors detailed in this section is listed in Table 3.

### 7.4. Treatment

Treatment of *Candida* spp. involves prompt initiation of antifungal therapy with echinocandins, azoles, and amphotericin B formulations (Pappas et al., 2016). The echinocandin group includes caspofungin, anidulafungin, and micafungin, all of which are noncompetitive inhibitors of the production of BDG (Denning, 2003). Given their broad-spectrum efficacy against *Candida* spp., echinocandins are frequently used to treat candidemia and invasive candidiasis (Pappas et al., 2016). In fact, initial antifungal therapy with fluconazole has been found an independent predictor of mortality elevating the risk death by roughly 200% in a cohort study in Italy (Pieralli et al., 2021). However, corroboration of this effect was inconclusive in a subsequent independent cohort study conducted in France, which compared echinocandins and azoles as first-line antifungal therapy without reaching significance in multivariate analyses (Bienvenu et al., 2020). Aside from *Candida auris*, antifungal resistance is systematically led by non-*albicans* species in China (Zhang et al., 2020; Liu et al., 2021), South Korea (Kwon et al., 2021), Turkey (Guner Ozenen et al., 2023), Italy (Mazzanti et al., 2021), Thailand (Ngamchokwathana et al., 2021), and Saudi Arabia (Al-Dorzi et al., 2018), out of which, *C. tropicalis* represents the major contributor. Data on resistance/susceptibility would appear to draw a trend of inverse sensitivity between compounds of the echinocandin group and fluconazole, with *C. albicans* displaying higher figures of resistance to echinocandin compounds than non-*albicans* species and lower figures of resistance to azole compounds than non-*albicans* species (Al-Dorzi et al., 2018; Jung et al., 2020; Guner Ozenen et al., 2023). Nevertheless, it has been reported that empirical administration of high-dose liposomal amphotericin B (L-AmB) is associated with better management of fungal invasiveness, less ICU-acquired candidemia, less need for an antifungal agent additional to L-AmB, and ultimately a reduction in ICU mortality, emphasizing the feasibility and relative safety of a preemptive antifungal therapy strategy to combat bloodstream *Candida* colonization (Azoulay et al., 2017). Regardless of the advancements detailed insofar, this review emphasizes the knowledge gap in molecular factors of resistance for *Candida* species, particularly when compared to the bacterial agents of sepsis reviewed above.

### 7.5. *Candida auris* sepsis

*Candia auris* is a human pathogenic yeast first isolated in Japan in 2009 (Satoh et al., 2009) that has gained notoriety for its high potential for invasive BSI, high mortality rates, moderate preventability, difficult identification by conventional techniques, and its virtually complete resistance to azoles (Geremia et al., 2023). Prompted by the escalating number of countries worldwide reporting the detection of *C. auris* (Briano et al., 2022; Riera et al., 2022; Figure 1), along with similar concerns for other neglected non-bloodstream fungal infections, the WHO has issued its first global effort to systematically prioritize fungal pathogens, in which *C. auris* ranks as the second most threating fungus to human health [World Health Organization (WHO), 2022b]. For an impeccable and updated review on *C. auris* virulence factors, risk factors, and antifungal resistance, please consult review by Geremia et al. (2023). Notwithstanding the growing number of studies describing its many facets, in pragmatic terms what concerns about *C. auris* sepsis is its often misclassification [as *C. famata*, *C. haemulonii*, or *Rhodotorula glutinis* (Kathuria et al., 2015)] and its pattern of multidrug resistance (Gómez-Gaviria et al., 2023).

Because host response to sepsis is inextricably canonical and *C. auris* virulence is considered intrinsically low (Geremia et al., 2023), the strong points for *C. auris* clinical management are then relegated to source control (Hinrichs et al., 2022) and drug delivery. With environmental source control (Akinbobola et al., 2023) falling partially outside of the scope of clinical management and efforts for nosocomial containment being not dissimilar to other fungal agents, a cardinal condition for source control is then the ability for detection. If the resources for accurate and routinely detection are limited, then emphasis is shifted toward drug delivery. With resistance to current drugs on the rise (Shastri et al., 2020; Briano et al., 2022), measurement of clinical management outcomes is then shifted from hospital discharge toward mortality. With information on specific *C. auris*-case fatality rates being scarce (Geremia et al., 2023), the extent of mortality then falls under the domain of speculation. Thus, we reckon that increasing access to detection and encouraging the appraisal of adverse outcomes are crucial milestones to tackle *C. auris*, particularly in locations where resources and logistics for diagnosis are limited, and consequently supporting the measures taken by the WHO [World Health Organization (WHO), 2022b], which stem from the precautionary principle (Goldstein, 2001). Although considerable steps have been taken very recently toward elucidating its pathobiology using mouse models (Wurster et al., 2022) and improving detection by employing advanced mathematical models (Garcia-Bustos et al., 2020), the knowledge gap about this emerging pathogen is systematic and efforts to contain it require a multidisciplinary approach.

## 8. Conclusion

Sepsis represents a topic with a high level of complexity that extends beyond patient management and clinical efforts for containment. In this multidisciplinary inter-collaborative scenario, curation of selected evidence is paramount to guide transversal action. We recognize the inherent limitations of *in vitro* studies, the controversy surrounding the verisimilitude of administering LPS (Osuchowski et al., 2018) or using cecal ligation and puncture (CLP) (Deutschman et al., 2022) as animal models of sepsis, as well as the impossibility of calculating factual figures of incidence and mortality worldwide (Rudd et al., 2020). Nevertheless, we value them as steps toward an integrated effort to tackle complexity straightforward and comprehensively. Thus, a list of novel approaches and potential therapeutical targets that should be considered for further research is presented in Table 4.

**Table 4:** Summary of potential therapeutical targets of interest for sepsis detailed in this review.

Approach/target	Mechanism	Reference
*E. coli*		
Bacterial cell membrane *ompA*	Deletion limits vascular permeability by reducing endothelial apoptosis and VE-Cadherin downregulation.	McHale et al. (2018)
*K. pneumoniae*		
Mushroom-derived β-glucans	Reduces bacterial load and improves physiological outcomes through dectin-1.	Masterson et al. (2020)
Bergenin monohydrate	Reduces bacterial load and improves physiological outcomes by reducing ROS production, increasing cell viability, increasing levels of SOD and GSH, reducing bacterial load, reducing levels of IL-6, IL-1β, PGE2, and TNF-α, reducing MDA formation, reducing MPO content, and reducing lung leukocyte infiltration.	Tang et al. (2021)
Inorganic compound AS101	Reduces bacterial load and improves survival by modulating apoptosis and inflammation.	Yang et al. (2021)
Membrane receptor TRAIL	Reduces bacterial load and improves survival by modulating apoptosis and inflammation.	Chen et al. (2019)
Adipose-derived mesenchymal stem cells	Reduces bacterial load and improves physiological outcomes by reducing levels of TNF-α, IL-1β, and IL-6, and immune infiltration in the lung.	Perlee et al. (2019)
Red blood cell membrane-coated PLGA nanoparticles	Reduces bacterial load and improves survival by reducing levels of TNF-α, IL-1β, and IL-6.	Liu et al. (2022)
Anti-K1-CPS antibodies	Reduces bacterial load and improves survival by promoting bacterial phagocytosis by Kupffer cells.	Diago-Navarro et al. (2017)
Blue light	Reduces bacterial load and improves survival through activation of neural circuit signaling through a cholinergic anti-inflammatory pathway	Griepentrog et al. (2020)
Supplementation of SCFA	Reduces bacterial load and improves survival by increasing macrophage phagocytic capacity and pulmonary levels of IL-6 and TNF-α.	Wu T. et al. (2020)
Acute phase protein PTX3	Reduces bacterial load and improves survival by inducing expression of TNF-α, IL-1β, IL-6, and CXCL-1, limiting MPO levels and leukocyte counts, and protecting from tissue hemorrhage.	Asgari et al. (2021)
Transcription factor HIF1α	Reduces bacterial load and improves physiological outcomes by limiting levels of pro-inflammatory cytokines while enhancing the release of IL-10 in the lung.	Otto et al. (2021)
*S. aureus*		
Antiplatelet drug ticagrelor	Reduces bacterial load and improves survival by extending protection from thrombocytopenia and protecting from organ damage.	Sun et al. (2021)
*P. aeruginosa*		
Regulator of α-ketoglutarate transport *MifR*	Deletion improves survival by limiting leukocyte infiltration, TNF-α, IL-6, and IL-1β production, NLRP3 inflammasome activation, and tissue damage.	Xiong et al. (2022)
Extracellular matrix degrading heparinase *hepP*	Deletion reduces bacterial load and improves survival.	Dzvova et al. (2018)
Endogenous H_2_S	Improves survival by enhancing neutrophil recruitment and phagocytic activity.	Renieris et al. (2021)
Peptidylarginine deiminase PAD2	Knockout mice exhibit reduced bacterial load and improved survival by reducing caspase-1-dependent pyroptosis in macrophages.	Wu W. et al. (2020)
Anti-PilQ-PilA DSL antibodies	Reduces bacterial load and improves survival in combination with antibiotics.	Zahedi bialvaei et al. (2021b)
PNM and 14–1 phage	Reduces bacterial load and shows successful in experimental therapy.	van Nieuwenhuyse et al. (2022)
*S. pyogenes*		
Non-canonical Tyr-phosphatase M5005_Spy_1476	Deletion reduces bacterial load and improves survival.	Kant and Pancholi (2021)
Predicted gene spy1343	Deletion reduces survival.	Sitkiewicz and Musser (2017)
Vascular endothelial growth factor	Administration improves survival.	Lu et al. (2022)
Pattern recognition receptor TLR13	Knockout mice exhibit limited macrophage IL-6 and NO_2_ production.	Hafner et al. (2019)

CXCL-1, CXC motif chemokine ligand 1; DSL, C-terminal disulfide loop; GSH, glutathione; HIF1α, hypoxia inducible factor 1 subunit α; IL, interleukin; K1-CPS, K1 serotype capsule polysaccharide; MDA, malondialdehyde; MPO, myeloperoxidase; NLRP3, NOD-like receptor family pyrin domain-containing 3; PAD2, peptidyl arginine deiminase type 2; PGE2, prostaglandin E2; PLGA, poly(lactic-co-glycolic acid); PTX3, pentraxin 3; ROS, reactive oxygen species; SCFA, short-chain fatty acids; SOD, superoxide dismutase; TLR13, toll-like receptor 13; TRAIL, tumor necrosis factor-related apoptosis-inducing ligand.

Transversal diversity of microorganism presentation in sepsis, from coinfection with multiple species to coexistence of multiple phylogroups, inextricably limits research conducted under the single-microorganism/single-strain approach right from its inception, for its reductionist nature. Notwithstanding the value in this frequent practice, this review makes clear that results emanating from integrative studies not only have the upper hand for describing reality but also are one step closer to clinical translation. An example of this is a very recent report identifying patterns of sepsis progression for most of the microorganisms described in this review using state-of-the-art genomics, transcriptomics, proteomics, and metabolomics, to make phylogenetic-oriented descriptions (Mu et al., 2023).

Indubitably, multidisciplinary advancement in basic, translational, and clinical research, is key to make progress filling the gap acknowledged by the World Health Organization (WHO) (2017) and endured yearly by millions around the globe.

## Author contributions

All authors listed have made a substantial, direct, and intellectual contribution to the work, and have approved it for publication.

## Funding

This study was funded by Agencia Nacional de Investigación y Desarrollo de Chile (ANID) through Fondo Nacional de Desarrollo Científico y Tecnológico (FONDECYT) grants 3220565 (SG), 1191300 (CR), and 11200764 (FM-G) and The Millennium Institute on Immunology and Immunotherapy/ANID – Millennium Science Initiative Program – ICM-ANID “ICN2021_045”: Millennium Institute on Immunology and Immunotherapy (Program ICM-ANID “ICN2021_045”) (SB, AK, FS, CR, and FM-G).

## Conflict of interest

The authors declare that the research was conducted in the absence of any commercial or financial relationships that could be construed as a potential conflict of interest.

## Publisher’s note

All claims expressed in this article are solely those of the authors and do not necessarily represent those of their affiliated organizations, or those of the publisher, the editors and the reviewers. Any product that may be evaluated in this article, or claim that may be made by its manufacturer, is not guaranteed or endorsed by the publisher.

## Glossary

APACHE II: Acute physiology and chronic health evaluation II
AUC: Area under the curve
AUROC: Area under the receiver operating characteristic
BDG: (1,3)-β-D-glucan
BSI: Bloodstream infection
COVID-19: Coronavirus disease 2019
CPS: Capsule polysaccharide
ECMO: Extracorporeal membrane oxygenation
ExPEC: Extraintestinal *E. coli*
UPEC: Uropathogenic *E. coli*
GSH: Glutathione
HR: Hazard ratio
HIF: Hypoxia inducible factor
hvKp: Hypervirulent *K. pneumoniae*
ICU: Intensive care unit
IL: Interleukin
LPS: Lipopolysaccharide
MRSA: Methicillin-resistant *S. aureus*
MSSA: Methicillin-sensitive *S. aureus*
MDR: Multi-drug resistant
NLRP3: NOD-like receptor family pyrin domain-containing 3
OR: Odds ratio
PVL: Panton–valentine leukocidin
PAD2: Peptidyl arginine deiminase type 2
PLGA: Poly (lactic-co-glycolic acid)
PGE2: Prostaglandin E2
ROS: Reactive oxygen species
SARS-CoV-2: Severe acute respiratory syndrome coronavirus 2
SPATE: Serine protease autotransporters of Enterobacteriaceae
SCFA: Short-chain fatty acid
SOD: Superoxide dismutase
SOFA: Sepsis organ failure assessment
TSS: Toxic shock syndrome
TNF: Tumor necrosis factor
VEGF: Vascular endothelial growth factor
WHO: World Health Organization
XDR: Extensively-drug resistant
XLA: X-linked agammaglobulinemia

## References

[ref1] Abreu A. G. Fraga T. R. Granados Martínez A. P. Kondo M. Y. Juliano M. A. Juliano L.. (2015). The serine protease pic from Enteroaggregative *Escherichia coli* mediates immune evasion by the direct cleavage of complement proteins. J. Infect. Dis. 212, 106–115. doi: 10.1093/infdis/jiv013, PMID: 25583166

[ref2] Agnelli C. Bouza E. del Carmen Martínez-Jiménez M. Navarro R. Valerio M. Machado M.. (2019). Clinical relevance and prognostic value of persistently negative (1,3)-β-D-glucan in adults with Candidemia: A 5-year experience in a tertiary hospital. Clin. Infect. Dis. 70, 1925–1932. doi: 10.1093/cid/ciz555, PMID: 31680136

[ref3] Ahmad N. I. Yean C. Y. Foo P. C. Safiee A. W. M. Hassan S. A. (2020). Prevalence and association of Panton-valentine Leukocidin gene with the risk of sepsis in patients infected with methicillin resistant *Staphylococcus aureus*. J. Infect. Public Health 13, 1508–1512. doi: 10.1016/j.jiph.2020.06.018 32653480

[ref4] Akinbobola A. B. Kean R. Hanifi S. M. A. Quilliam R. S. (2023). Environmental reservoirs of the drug-resistant pathogenic yeast Candida auris. PLoS Pathog. 19:e1011268. doi: 10.1371/journal.ppat.1011268 37053164 PMC10101498

[ref5] Aldardeer N. F. Albar H. al-Attas M. Eldali A. Qutub M. Hassanien A.. (2020). Antifungal resistance in patients with Candidaemia: a retrospective cohort study. BMC Infect. Dis. 20:55. doi: 10.1186/s12879-019-4710-z, PMID: 31952505 PMC6969401

[ref6] Al-Dorzi H. M. Sakkijha H. Khan R. Aldabbagh T. Toledo A. Ntinika P.. (2018). Invasive candidiasis in critically ill patients: A prospective cohort study in two tertiary care centers. J. Intensive Care Med. 35, 542–553. doi: 10.1177/0885066618767835, PMID: 29628014 PMC7222290

[ref7] Alessandri F. Ceccarelli G. Migliara G. Baccolini V. Russo A. Marzuillo C.. (2023). High incidence of Candidemia in critically ill COVID-19 patients supported by Veno-venous extracorporeal membrane oxygenation: A retrospective study. J. Fungi 9:119. doi: 10.3390/jof9010119, PMID: 36675940 PMC9861971

[ref8] Al-Hasan M. N. Eckel-Passow J. E. Baddour L. M. (2012). Impact of healthcare-associated acquisition on community-onset gram-negative bloodstream infection: a population-based study. Eur. J. Clin. Microbiol. Infect. Dis. 31, 1163–1171. doi: 10.1007/s10096-011-1424-6 21983895 PMC3369543

[ref9] Alhazzani W. Møller M. H. Arabi Y. M. Loeb M. Gong M. N. Fan E.. (2020). Surviving Sepsis campaign: guidelines on the management of critically ill adults with coronavirus disease 2019 (COVID-19). Intensive Care Med. 46, 854–887. doi: 10.1007/s00134-020-06022-5, PMID: 32222812 PMC7101866

[ref10] Al-Musawi T. S. Alkhalifa W. A. Alasaker N. A. Rahman J. U. Alnimr A. M. (2021). A seven-year surveillance of Candida bloodstream infection at a university hospital in KSA. J. Taibah Univ. Med. Sci. 16, 184–190. doi: 10.1016/j.jtumed.2020.12.002 33897322 PMC8046963

[ref11] Ang S. H. Petrick P. Shamsul A. S. Ramliza R. Kori N. Lau C. L. (2022). The risk factors for complications and survival outcomes of *Klebsiella pneumoniae* Bacteraemia in hospital Canselor Tuanku Muhriz Universiti Kebangsaan Malaysia. Med. J. Malays. 77, 440–445. 35902933

[ref12] Arora H. S. Khan H. Ailumerab H. Natarajan G. Meert K. Salimnia H.. (2023). A tale of two intensive care units (ICUs): baseline *Staphylococcus aureus* colonization and mupirocin susceptibility in neonatal and pediatric patients requiring intensive care. Infect. Control. 44, 447–452. doi: 10.1017/ice.2022.96, PMID: 35450544 PMC10015265

[ref13] Asai N. Ohashi W. Sakanashi D. Suematsu H. Kato H. Hagihara M.. (2021). Combination of sequential organ failure assessment (SOFA) score and Charlson comorbidity index (CCI) could predict the severity and prognosis of candidemia more accurately than the acute physiology, age, chronic health evaluation II (APACHE II) score. BMC Infect. Dis. 21:77. doi: 10.1186/s12879-020-05719-8, PMID: 33451284 PMC7811217

[ref14] Asgari F. Supino D. Parente R. Polentarutti N. Stravalaci M. Porte R.. (2021). The long Pentraxin PTX3 controls *Klebsiella Pneumoniae* severe infection. Front. Immunol. 12:666198. doi: 10.3389/fimmu.2021.666198, PMID: 34093560 PMC8173212

[ref15] Azoulay E. Timsit J.-F. Lautrette A. Legriel S. Max A. Ruckly S.. (2017). Weekly high-dose liposomal amphotericin B (L-AmB) in critically ill septic patients with multiple Candida colonization: the AmBiDex study. PLoS One 12:e0177093. doi: 10.1371/journal.pone.0177093, PMID: 28531175 PMC5439673

[ref16] Babiker A. Li X. Lai Y. L. Strich J. R. Warner S. Sarzynski S.. (2021). Effectiveness of adjunctive clindamycin in β-lactam antibiotic-treated patients with invasive β-haemolytic streptococcal infections in US hospitals: a retrospective multicentre cohort study. Lancet Infect. Dis. 21, 697–710. doi: 10.1016/s1473-3099(20)30523-5, PMID: 33333013 PMC8084921

[ref17] Bachmann N. L. Katouli M. Polkinghorne A. (2015). Genomic comparison of translocating and non-translocating *Escherichia coli*. PLoS One 10:e0137131. doi: 10.1371/journal.pone.0137131 26317913 PMC4552563

[ref18] Bai A. D. Bonares M. J. Thrall S. Bell C. M. Morris A. M. (2020). Presence of urinary symptoms in bacteremic urinary tract infection: a retrospective cohort study of *Escherichia coli* bacteremia. BMC Infect. Dis. 20:781. doi: 10.1186/s12879-020-05499-1 33081714 PMC7576869

[ref19] Bartie K. L. Williams D. W. Wilson M. J. Potts A. J. C. Lewis M. A. O. (2004). Differential invasion of *Candida albicans* isolates in an in vitro model of oral candidosis. Oral Microbiol. Immunol. 19, 293–296. doi: 10.1111/j.1399-302x.2004.00155.x 15327640

[ref20] Bartoletti M. Rinaldi M. Pasquini Z. Scudeller L. Piano S. Giacobbe D. R.. (2021). Risk factors for candidaemia in hospitalized patients with liver cirrhosis: a multicentre case–control–control study. CMI 27, 276–282. doi: 10.1016/j.cmi.2020.04.030, PMID: 32360775

[ref21] Bassetti M. Castaldo N. Cattelan A. Mussini C. Righi E. Tascini C.. (2019). Ceftolozane/tazobactam for the treatment of serious *Pseudomonas aeruginosa* infections: a multicentre nationwide clinical experience. Antimicrob. Agents Annu. 53, 408–415. doi: 10.1016/j.ijantimicag.2018.11.001, PMID: 30415002

[ref22] Bassetti M. Righi E. del Giacomo P. Sartor A. Ansaldi F. Trucchi C.. (2018). Predictors of mortality with *Staphylococcus aureus* bacteremia in elderly adults. J. Am. Geriatr. Soc. 66, 1284–1289. doi: 10.1111/jgs.15391, PMID: 29664994

[ref23] Bassetti M. Vena A. Meroi M. Cardozo C. Cuervo G. Giacobbe D. R.. (2020). Factors associated with the development of septic shock in patients with candidemia: a post hoc analysis from two prospective cohorts. Crit. Care 24:117. doi: 10.1186/s13054-020-2793-y, PMID: 32216822 PMC7099832

[ref24] Battistolo J. Glampedakis E. Damonti L. Poissy J. Grandbastien B. Kalbermatter L.. (2021). Increasing morbidity and mortality of candidemia over one decade in a Swiss university hospital. Mycoses 64, 1512–1520. doi: 10.1111/myc.13376, PMID: 34587318 PMC9298218

[ref25] Belo V. A. Pereira J. A. Souza S. F. D. Tana F. d. L. Pereira B. P. Lopes D. d. O.. (2021). The role of IL-10 in immune responses against *Pseudomonas aeruginosa* during acute lung infection. Cell Tissue Res. 383, 1123–1133. doi: 10.1007/s00441-020-03308-4, PMID: 33165659

[ref26] Bhardwaj N. K. Khera D. Gupta N. Singh K. (2017). Disseminated *Pseudomonas aeruginosa* sepsis as presenting diagnosis of X-linked agammaglobulinaemia in a previously well 16-month-old child. Bmj Case Rep. 2017, 1–4. doi: 10.1136/bcr-2017-221006 PMC5624027 28851726

[ref27] Bienvenu A.-L. Pradat P. Guerin C. Aubrun F. Fellahi J.-L. Friggeri A.. (2020). Evaluation of first-line therapies for the treatment of candidemia in ICU patients: A propensity score analysis. Int. J. Infect. Dis. 93, 15–21. doi: 10.1016/j.ijid.2020.01.037, PMID: 31982622

[ref28] Birlutiu V. Birlutiu R. M. Baicu M. Iancu G. M. (2019). A case report of double etiology of ecthyma gangrenosum. Medicine 98:e15651. doi: 10.1097/md.0000000000015651 31096489 PMC6531273

[ref29] Biscaye S. Demonchy D. Afanetti M. Dupont A. Haas H. Tran A. (2017). Ecthyma gangrenosum, a skin manifestation of *Pseudomonas aeruginosa* sepsis in a previously healthy child. Medicine 96:e5507. doi: 10.1097/md.0000000000005507 28079790 PMC5266152

[ref30] Bläckberg A. Svedevall S. Lundberg K. Nilson B. Kahn F. Rasmussen M. (2022). Time to blood culture positivity: an independent predictor of mortality in *Streptococcus Pyogenes* bacteremia. Open forum. Infect. Dis. Ther. 9:ofac163. doi: 10.1093/ofid/ofac163 PMC9126491 35615297

[ref31] Blagden S. Watts V. Verlander N. Q. Pegorie M. (2020). Invasive group A streptococcal infections in north West England: epidemiology, risk factors and fatal infection. Public Health 186, 63–70. doi: 10.1016/j.puhe.2020.06.007 32784097

[ref32] Bongomin F. Gago S. Oladele R. Denning D. (2017). Global and multi-National Prevalence of fungal diseases—estimate precision. J. Fungi 3:57. doi: 10.3390/jof3040057 PMC5753159 29371573

[ref33] Bonten M. Johnson J. R. Biggelaar A. H. J. van den Georgalis L. Geurtsen J. Palacios P. I. de. (2020). Epidemiology of *Escherichia coli* bacteremia: A systematic literature review. Clin. Infect. Dis. 72, 1211–1219. doi: 10.1093/cid/ciaa210, PMID: 32406495

[ref34] Bouza E. Burillo A. Muñoz P. Guinea J. Marín M. Rodríguez-Créixems M. (2013). Mixed bloodstream infections involving bacteria and Candida spp. J. Antimicrob. Chemother. 68, 1881–1888. doi: 10.1093/jac/dkt099 23535881

[ref35] Boyle-Vavra S. Li X. Alam M. T. Read T. D. Sieth J. Cywes-Bentley C.. (2015). USA300 and USA500 clonal lineages of *Staphylococcus aureus* do not produce a capsular polysaccharide due to conserved mutations in thecap5Locus. MBio 6:e02585. doi: 10.1128/mbio.02585-14 25852165 PMC4453534

[ref36] Brammer J. Wolf G. Baliban S. M. Allen J. C. Choi M. Kambouris A. R.. (2021). A nonlethal full-thickness flame burn produces a seroma beneath the forming eschar, thereby promoting *Pseudomonas aeruginosa* Sepsis in mice. J. Burn Care Res. 43, 792–801. doi: 10.1093/jbcr/irab195, PMID: 34739051 PMC9249144

[ref37] Briano F. Magnasco L. Sepulcri C. Dettori S. Dentone C. Mikulska M.. (2022). Candida auris Candidemia in critically ill, colonized patients: cumulative incidence and risk factors. Infect. Dis. Ther. 11, 1149–1160. doi: 10.1007/s40121-022-00625-9, PMID: 35404010 PMC8995918

[ref38] Brundage J. F. (2006). Interactions between influenza and bacterial respiratory pathogens: implications for pandemic preparedness. Lancet Infect. Dis. 6, 303–312. doi: 10.1016/s1473-3099(06)70466-2 16631551 PMC7106411

[ref39] Butt J. H. Fosbøl E. L. Gerds T. A. Iversen K. Bundgaard H. Bruun N. E.. (2020). Ticagrelor and the risk of *Staphylococcus aureus* bacteraemia and other infections. Eur. Heart J. 8, 13–19. doi: 10.1093/ehjcvp/pvaa099, PMID: 32750138

[ref40] Cebrero-Cangueiro T. Labrador-Herrera G. Pascual Á. Díaz C. Rodríguez-Baño J. Pachón J.. (2021). Efficacy of Fosfomycin and its combination with aminoglycosides in an experimental Sepsis model by Carbapenemase-producing *Klebsiella pneumoniae* clinical strains. Front. Med. 8:615540. doi: 10.3389/fmed.2021.615540, PMID: 33842497 PMC8033020

[ref41] Chang D. Sharma L. Cruz C. S. D. Zhang D. (2021). Clinical epidemiology, risk factors, and control strategies of *Klebsiella pneumoniae* infection. Front. Microbiol. 12:750662. doi: 10.3389/fmicb.2021.750662 34992583 PMC8724557

[ref42] Chapelet G. Boureau A. S. Dylis A. Herbreteau G. Corvec S. Batard E.. (2017). Association between dementia and reduced walking ability and 30-day mortality in patients with extended-spectrum beta-lactamase-producing *Escherichia coli* bacteremia. Eur. J. Clin. Microbiol. Infect. Dis. 36, 2417–2422. doi: 10.1007/s10096-017-3077-6, PMID: 28801698

[ref43] Chen Y.-F. Chen G.-Y. Chang C.-H. Su Y.-C. Chen Y.-C. Jiang Y.. (2019). TRAIL encapsulated to polypeptide-crosslinked nanogel exhibits increased anti-inflammatory activities in *Klebsiella pneumoniae*-induced sepsis treatment. Mater. Sci. Eng. C 102, 85–95. doi: 10.1016/j.msec.2019.04.023, PMID: 31147057

[ref44] Chen Y. N. Hsu J.-F. Chu S.-M. Lai M.-Y. Lin C. Huang H.-R.. (2022). Clinical and microbiological characteristics of neonates with Candidemia and impacts of therapeutic strategies on the outcomes. J. Fungi 8:465. doi: 10.3390/jof8050465, PMID: 35628721 PMC9148079

[ref45] Chen W. Lian J. Ye J. Mo Q. Qin J. Hong G.. (2017). Ethyl pyruvate reverses development of *Pseudomonas aeruginosa* pneumonia during sepsis-induced immunosuppression. Int. Immunopharmacol. 52, 61–69. doi: 10.1016/j.intimp.2017.08.024, PMID: 28863323

[ref46] Chen I. R. Lin S.-N. Wu X.-N. Chou S.-H. Wang F.-D. Lin Y.-T. (2022). Clinical and microbiological characteristics of Bacteremic pneumonia caused by *Klebsiella pneumoniae*. Front Cell Infect Microbiol. 12:903682. doi: 10.3389/fcimb.2022.903682 35811668 PMC9259976

[ref47] Chen X.-C. Xu J. Wu D.-P. (2020). Clinical characteristics and implications of mixed candida/bacterial bloodstream infections in patients with hematological diseases. Eur. J. Clin. Microbiol. Infect. Dis. 39, 1445–1452. doi: 10.1007/s10096-020-03863-2 32170543

[ref48] Chochua S. Metcalf B. Li Z. Mathis S. Tran T. Rivers J.. (2022). Invasive group A streptococcal penicillin binding protein 2× variants associated with reduced susceptibility to β-lactam antibiotics in the United States, 2015–2021. ASM J. CD 66:e0080222. doi: 10.1128/aac.00802-22, PMID: 35969070 PMC9487518

[ref49] Choi M. Hegerle N. Nkeze J. Sen S. Jamindar S. Nasrin S.. (2020). The diversity of lipopolysaccharide (O) and capsular polysaccharide (K) antigens of invasive *Klebsiella pneumoniae* in a multi-country collection. Front. Microbiol. 11:1249. doi: 10.3389/fmicb.2020.01249, PMID: 32595624 PMC7303279

[ref50] Cienfuegos-Gallet A. V. Zhou Y. Ai W. Kreiswirth B. N. Yu F. Chen L. (2022). Multicenter genomic analysis of Carbapenem-resistant *Klebsiella pneumoniae* from bacteremia in China. Microbiol. Spectr. 10, e02290–e02221. doi: 10.1128/spectrum.02290-21 35230130 PMC9045280

[ref51] Clancy C. J. Kalil A. C. Fowler V. G. Ghedin E. Kolls J. K. Nguyen M. H. (2014). Emerging and resistant infections. Ann. Am. Thorac. Soc. 11, S193–S200. doi: 10.1513/annalsats.201402-069pl 25148425 PMC4200571

[ref52] Clermont O. Dixit O. V. A. Vangchhia B. Condamine B. Dion S. Bridier-Nahmias A.. (2019). Characterization and rapid identification of phylogroup G in *Escherichia coli*, a lineage with high virulence and antibiotic resistance potential. Environ. Microbiol. 21, 3107–3117. doi: 10.1111/1462-2920.14713, PMID: 31188527

[ref53] Coggon C. F. Jiang A. Goh K. G. K. Henderson I. R. Schembri M. A. Wells T. J. (2018). A novel method of serum resistance by *Escherichia coli* that causes Urosepsis. MBio 9, e00918–e00920. doi: 10.1128/mbio.00920-18 PMC6020292 29946047

[ref54] Coombs G. W. Daley D. A. Yee N. W. Shoby P. Mowlaboccus S. (2022). Australian group on antimicrobial resistance (AGAR) Australian *Staphylococcus aureus* Sepsis outcome Programme (ASSOP) annual report 2020. Commun. Dis. Intell. 46, 1–17. doi: 10.33321/cdi.2022.46.18 35469556

[ref55] Coppola P. E. Gaibani P. Sartor C. Ambretti S. Lewis R. E. Sassi C.. (2020). Ceftolozane-Tazobactam treatment of Hypervirulent multidrug resistant *Pseudomonas aeruginosa* infections in neutropenic patients. Microorganisms 8:2055. doi: 10.3390/microorganisms8122055, PMID: 33371496 PMC7767535

[ref56] Corbella L. Boán J. San-Juan R. Fernández-Ruiz M. Carretero O. Lora D.. (2022). Effectiveness of ceftazidime-avibactam for the treatment of infections due to *Pseudomonas aeruginosa*. Int. J. Antimicrob. Agents 59:106517. doi: 10.1016/j.ijantimicag.2021.106517 34990760

[ref57] Cusumano J. A. Dupper A. C. Malik Y. Gavioli E. M. Banga J. Berbel Caban A.. (2020). *Staphylococcus aureus* bacteremia in patients infected with COVID-19: A case series. Infect. Dis. Ther. 7:ofaa518. doi: 10.1093/ofid/ofaa518, PMID: 33269299 PMC7686656

[ref58] Damico V. Murano L. Margosio V. Ripamonti C. (2022). Co-infections among COVID-19 adult patients admitted to intensive care units: results from a retrospective study. Annu. DiIgiene Med. Prev. Comun. 35, 49–60. doi: 10.7416/ai.2022.2515 35195240

[ref59] Davis J. Turnidge J. Tong S. (2018). A large retrospective cohort study of cefazolin compared with flucloxacillin for methicillin-susceptible *Staphylococcus aureus* bacteraemia. Int. J. Antimicrob. Agent 52, 297–300. doi: 10.1016/j.ijantimicag.2018.02.013 29499317

[ref60] Lastours V. de Laouénan C. Royer G. Carbonnelle E. Lepeule R. Esposito-Farèse M.. (2020). Mortality in *Escherichia coli* bloodstream infections: antibiotic resistance still does not make it. J. Antimicrob. Chemother. 75, 2334–2343. doi: 10.1093/jac/dkaa161, PMID: 32417924

[ref61] Delaloye J. Calandra T. (2014). Invasive candidiasis as a cause of sepsis in the critically ill patient. Virulence 5, 161–169. doi: 10.4161/viru.26187 24157707 PMC3916370

[ref62] DeMuri G. P. Sterkel A. K. Kubica P. A. Duster M. N. Reed K. D. Wald E. R. (2017). Macrolide and clindamycin resistance in group a streptococci isolated from children with pharyngitis. Pediatr. Infect. Dis. J. 36, 342–344. doi: 10.1097/inf.0000000000001442 27902646

[ref63] Denning D. W. (2003). Echinocandin antifungal drugs. Lancet 362, 1142–1151. doi: 10.1016/s0140-6736(03)14472-8 14550704

[ref64] Desvaux M. Dalmasso G. Beyrouthy R. Barnich N. Delmas J. Bonnet R. (2020). Pathogenicity factors of Genomic Islands in intestinal and Extraintestinal *Escherichia coli*. Front. Microbiol. 11:2065. doi: 10.3389/fmicb.2020.02065 33101219 PMC7545054

[ref65] Deutschman C. S. Leisman D. E. Taylor M. D. (2022). Adrenergic immune effects: is Beta the enemy of good?*. Crit. Care Med. 50, 1415–1418. doi: 10.1097/ccm.0000000000005524 35984059

[ref66] di Domenico E. G. Marchesi F. Cavallo I. Toma L. Sivori F. Papa E.. (2021). The impact of bacterial biofilms on end-organ disease and mortality in patients with hematologic malignancies developing a bloodstream infection. Microbiol. Spectr. 9:e0055021. doi: 10.1128/spectrum.00550-21, PMID: 34406812 PMC8552682

[ref67] Diago-Navarro E. Calatayud-Baselga I. Sun D. Khairallah C. Mann I. Ulacia-Hernando A.. (2017). Antibody-based immunotherapy to treat and prevent infection with Hypervirulent *Klebsiella pneumoniae*. Clin. Vaccine Immunol. 24, 1–10. doi: 10.1128/cvi.00456-16, PMID: 27795303 PMC5216427

[ref68] Díaz J. M. Dozois C. M. Avelar-González F. J. Hernández-Cuellar E. Pokharel P. Santiago A. S. de. (2020). The Vacuolating autotransporter toxin (vat) of *Escherichia coli* causes cell cytoskeleton changes and produces non-lysosomal vacuole formation in bladder epithelial cells. Front. Microbiol. 10,:299. doi: 10.3389/fcimb.2020.00299, PMID: 32670893 PMC7332727

[ref69] Diekema D. J. Hsueh P.-R. Mendes R. E. Pfaller M. A. Rolston K. V. Sader H. S.. (2019). The microbiology of bloodstream infection: 20-year trends from the SENTRY antimicrobial surveillance program. ASM J. CD 63, e00355–e00419. doi: 10.1128/aac.00355-19, PMID: 31010862 PMC6591610

[ref70] Doğan Ö. Yeşilkaya A. Menekşe Ş. Güler Ö. Karakoç Ç. Çınar G.. (2020). Effect of initial antifungal therapy on mortality among patients with bloodstream infections with different Candida species and resistance to antifungal agents: A multicentre observational study by the Turkish fungal infections study group. Antimicrob. Agents Annu. 56:105992. doi: 10.1016/j.ijantimicag.2020.105992, PMID: 32335275

[ref71] Donnelly J. P. Chen S. C. Kauffman C. A. Steinbach W. J. Baddley J. W. Verweij P. E.. (2019). Revision and update of the consensus definitions of invasive fungal disease from the European Organization for Research and Treatment of Cancer and the mycoses study group education and research consortium. Clin. Infect. Dis. 71, 1367–1376. doi: 10.1093/cid/ciz1008, PMID: 31802125 PMC7486838

[ref72] Dzvova N. Colmer-Hamood J. A. Griswold J. A. Hamood A. N. (2018). Heparinase is essential for *Pseudomonas aeruginosa* virulence during thermal injury and infection. Infect. Immun. 86, 1–13. doi: 10.1128/iai.00755-17 PMC5736813 29061710

[ref73] Eichenberger E. M. Dagher M. Ruffin F. Park L. Hersh L. Sivapalasingam S.. (2020). Complement levels in patients with bloodstream infection due to *Staphylococcus aureus* or gram-negative bacteria. Eur. J. Clin. Microbiol. Infect. Dis. 39, 2121–2131. doi: 10.1007/s10096-020-03955-z, PMID: 32621149 PMC7334117

[ref74] Eisi H. Ibraheem S. Hisham T. al-Harbi A. Saidy K. Ali I.. (2022). Risk factors and outcomes of deep tissue Candida invasion in neonates with invasive candidiasis. Mycoses 65, 110–119. doi: 10.1111/myc.13395, PMID: 34780084

[ref75] Erayil S. E. Palzer E. Kline S. (2022). An evaluation of risk factors for *Staphylococcus aureus* colonization in a pre-surgical population. Access Microbiol. 4:000316. doi: 10.1099/acmi.0.000316 35252754 PMC8895606

[ref76] Escobar-Páramo P. Clermont O. Blanc-Potard A.-B. Bui H. Bouguénec C. L. Denamur E. (2004). A specific genetic background is required for acquisition and expression of virulence factors in *Escherichia coli*. Mol. Biol. Evol. 21, 1085–1094. doi: 10.1093/molbev/msh118 15014151

[ref77] Esparcia A. Madrazo M. Alberola J. López-Cruz I. Eiros J. M. Nogueira J. M.. (2019). Community-onset *Pseudomonas aeruginosa* urinary sepsis in elderly people: predictive factors, adequacy of empirical therapy and outcomes. Int. J. Clin. Pract. 73:e13425. doi: 10.1111/ijcp.13425, PMID: 31573737

[ref78] Evans L. Rhodes A. Alhazzani W. Antonelli M. Coopersmith C. M. French C.. (2021). Surviving Sepsis campaign: international guidelines for Management of Sepsis and Septic Shock 2021. Crit. Care Med. 49, e1063–e1143. doi: 10.1097/ccm.0000000000005337, PMID: 34605781

[ref79] Ferreira M. Santos M. Rodrigues J. Diogo C. Resende C. Baptista C.. (2023). Epidemiology of bacteremia in a pediatric population – A 10-year study. Enferm. Infecc. Microbiol. Clin. 41, 85–91. doi: 10.1016/j.eimce.2021.06.006, PMID: 36759058

[ref80] Follador R. Heinz E. Wyres K. L. Ellington M. J. Kowarik M. Holt K. E.. (2016). The diversity of *Klebsiella pneumoniae* surface polysaccharides. Microb. Genom. 2:e000073. doi: 10.1099/mgen.0.000073, PMID: 28348868 PMC5320592

[ref81] Freire C. A. Santos A. C. M. Pignatari A. C. Silva R. M. Elias W. P. (2020). Serine protease autotransporters of Enterobacteriaceae (SPATEs) are largely distributed among *Escherichia coli* isolated from the bloodstream. Braz. J. Microbiol. 51, 447–454. doi: 10.1007/s42770-020-00224-1 31965549 PMC7203317

[ref82] Freire C. A. Silva R. M. Ruiz R. C. Pimenta D. C. Bryant J. A. Henderson I. R.. (2022). Secreted autotransporter toxin (sat) mediates innate immune system evasion. Front. Immunol. 13:844878. doi: 10.3389/fimmu.2022.844878, PMID: 35251044 PMC8891578

[ref83] Fröding I. Hasan B. Sylvin I. Coorens M. Nauclér P. Giske C. G. (2020). Extended-Spectrum-β-lactamase- and plasmid AmpC-producing *Escherichia coli* causing community-onset bloodstream infection: Association of Bacterial Clones and Virulence Genes with septic shock, source of infection, and recurrence. Antimicrob. Agents Chem. 64, 1–17. doi: 10.1128/aac.02351-19 PMC7526827 32423949

[ref84] Gaensbauer J. T. Birkholz M. Smit M. A. Garcia R. Todd J. K. (2018). Epidemiology and clinical relevance of toxic shock syndrome in US children. Pediatr. Infect. Dis. J. 37, 1223–1226. doi: 10.1097/inf.0000000000002002 29601458

[ref85] Gajdács M. Ábrók M. Lázár A. Burián K. (2020). Beta-Haemolytic group A, C and G streptococcal infections in southern Hungary: A 10-year population-based retrospective survey (2008–2017) and a review of the literature. Infect Drug Resist 13, 4739–4749. doi: 10.2147/idr.s279157 33408489 PMC7781025

[ref86] Garcia-Bustos V. Salavert M. Ruiz-Gaitán A. C. Cabañero-Navalon M. D. Sigona-Giangreco I. A. Pemán J. (2020). A clinical predictive model of candidaemia by Candida auris in previously colonized critically ill patients. Clin. Microbiol. Infect. 26, 1507–1513. doi: 10.1016/j.cmi.2020.02.001 32061792

[ref87] Garcia-Vidal C. Sanjuan G. Moreno-García E. Puerta-Alcalde P. Garcia-Pouton N. Chumbita M.. (2021). Incidence of co-infections and superinfections in hospitalized patients with COVID-19: a retrospective cohort study. CMI 27, 83–88. doi: 10.1016/j.cmi.2020.07.041, PMID: 32745596 PMC7836762

[ref88] Gatica S. Villegas V. Vallejos A. Olivares P. Aballai V. Lagos-Meza F.. (2020). TRPM7 mediates kidney injury, endothelial hyperpermeability and mortality during endotoxemia. Lab. Investig. 100, 234–249. doi: 10.1038/s41374-019-0304-z, PMID: 31444399

[ref01] GBD 2019 Antimicrobial Resistance Collaborators. (2022). Global mortality associated with 33 bacterial pathogens in 2019: a systematic analysis for the Global Burden of Disease Study 2019. Lancet 400, 2221–2248. doi: 10.1016/s0140-6736(22)02185-7 36423648 PMC9763654

[ref89] Gebremicael M. N. Nuttall J. J. C. Tootla H. D. Khumalo A. Tooke L. Salie S.. (2023). Candida bloodstream infection among children hospitalised in three public-sector hospitals in the metro West region of Cape Town, South Africa. Infect. Dis. Ther. 23:67. doi: 10.1186/s12879-023-08027-z, PMID: 36737689 PMC9896677

[ref90] Gera K. McIver K. S. (2013). Laboratory growth and maintenance of *Streptococcus pyogenes* (the group A Streptococcus, GAS). Curr. Protoc. Microbiol. 30, 9D.2.1–9D.2.13. doi: 10.1002/9780471729259.mc09d02s30 PMC3920295 24510893

[ref91] Geremia N. Brugnaro P. Solinas M. Scarparo C. Panese S. (2023). Candida auris as an emergent public health problem: A current update on European outbreaks and cases. Health 11:425. doi: 10.3390/healthcare11030425 PMC9914380 36767000

[ref92] Giovannenze F. Murri R. Palazzolo C. Taccari F. Camici M. Spanu T.. (2021). Predictors of mortality among adult, old and the oldest old patients with bloodstream infections: an age comparison. Eur. J. Intern. Med. 86, 66–72. doi: 10.1016/j.ejim.2020.12.017, PMID: 33414015

[ref93] Glass E. L. Raoult D. Dubourg G. (2022). Snapshot of COVID-19 superinfections in Marseille hospitals: where are the common pathogens? Epidemiol. Infect. 150:e195. doi: 10.1017/s0950268822001704 36345840 PMC9744451

[ref94] Goldstein B. D. (2001). The precautionary principle also applies to public health actions. Am. J. Public Health 91, 1358–1361. doi: 10.2105/ajph.91.9.1358 11527755 PMC1446778

[ref95] Gómez-Gaviria M. Martínez-Álvarez J. A. Chávez-Santiago J. O. Mora-Montes H. M. (2023). Candida haemulonii complex and Candida auris: biology, virulence factors, immune response, and multidrug resistance. Infect Drug Resist 16, 1455–1470. doi: 10.2147/idr.s402754 36942024 PMC10024503

[ref96] González-Lara M. F. Torres-González P. Cornejo-Juárez P. Velázquez-Acosta C. Martinez-Gamboa A. Rangel-Cordero A.. (2017). Impact of inappropriate antifungal therapy according to current susceptibility breakpoints on Candida bloodstream infection mortality, a retrospective analysis. BMC Infect. Dis. 17:753. doi: 10.1186/s12879-017-2846-2, PMID: 29212442 PMC5719515

[ref97] Gorrie C. L. Mirceta M. Wick R. R. Edwards D. J. Thomson N. R. Strugnell R. A.. (2017). Gastrointestinal carriage is a Major reservoir of *Klebsiella pneumoniae* infection in intensive care patients. Clin. Infect. Dis. 65, 208–215. doi: 10.1093/cid/cix270, PMID: 28369261 PMC5850561

[ref98] Gouel-Cheron A. Swihart B. J. Warner S. Mathew L. Strich J. R. Mancera A.. (2022). Epidemiology of ICU-onset bloodstream infection: prevalence, pathogens, and risk factors among 150,948 ICU patients at 85 U.S. hospitals*. Crit. Care Med. 50, 1725–1736. doi: 10.1097/ccm.0000000000005662, PMID: 36190259 PMC10829879

[ref99] Greenberg J. A. Hrusch C. L. Jaffery M. R. David M. Z. Daum R. S. Hall J. B.. (2018). Distinct T-helper cell responses to *Staphylococcus aureus* bacteremia reflect immunologic comorbidities and correlate with mortality. Crit. Care 22:107. doi: 10.1186/s13054-018-2025-x, PMID: 29695270 PMC5916828

[ref100] Griepentrog J. E. Zhang X. Lewis A. J. Gianfrate G. Labiner H. E. Zou B.. (2020). Frontline science: rev-Erbα links blue light with enhanced bacterial clearance and improved survival in murine *Klebsiella pneumoniae* pneumonia. J. Leukoc. Biol. 107, 11–25. doi: 10.1002/jlb.4hi0519-155r, PMID: 31379019 PMC7456460

[ref101] Gröndahl-Yli-Hannuksela K. Beres S. B. Hyyryläinen H.-L. Kallonen T. Musser J. M. Vuopio J. (2021). Genetic evolution of invasive emm28 *Streptococcus pyogenes* strains and significant association with puerperal infections in young women in Finland. Clin. Microbiol. Infect. 27, 420–427. doi: 10.1016/j.cmi.2020.04.004 32289480 PMC7780161

[ref102] Guner Ozenen G. Sahbudak Bal Z. Avcu G. Ozkaya Yazici P. Karakoyun M. Metin D. Y.. (2023). Evaluation of candidemia in children at a university hospital: A retrospective cohort. Mycoses 66, 367–377. doi: 10.1111/myc.13564, PMID: 36597951

[ref103] Gupta E. Kumar S. Srivastava V. K. Saxena J. Siddiqui A. J. Mehta S.. (2022). Unravelling the differential host Immuno-inflammatory responses to Staphylococcus aureus and *Escherichia coli* infections in Sepsis. Vaccine 10:1648. doi: 10.3390/vaccines10101648, PMID: 36298513 PMC9610428

[ref104] Gupte A. Jyot J. Ravi M. Ramphal R. (2021). High pyocyanin production and non-motility of *Pseudomonas aeruginosa* isolates are correlated with septic shock or death in bacteremic patients. PLoS One 16:e0253259. doi: 10.1371/journal.pone.0253259 34115807 PMC8195364

[ref105] Guthridge I. Smith S. Law M. Binotto E. Hanson J. (2021). Efficacy and safety of intravenous Lincosamide therapy in methicillin-resistant *Staphylococcus aureus* bacteremia. Antimicrob. Agents Chem. 65, e00321–e00343. doi: 10.1128/aac.00343-21 PMC8370195 34125589

[ref106] Hafner A. Kolbe U. Freund I. Castiglia V. Kovarik P. Poth T.. (2019). Crucial role of nucleic acid sensing via endosomal toll-like receptors for the defense of *Streptococcus pyogenes* in vitro and in vivo. Front. Immunol. 10:198. doi: 10.3389/fimmu.2019.00198, PMID: 30846984 PMC6394247

[ref107] Hammer K. L. Justo J. A. Bookstaver P. B. Kohn J. Albrecht H. Al-Hasan M. N. (2017). Differential effect of prior β-lactams and fluoroquinolones on risk of bloodstream infections secondary to *Pseudomonas aeruginosa*. Diagn. Microbiol. Infect. Dis. 87, 87–91. doi: 10.1016/j.diagmicrobio.2016.09.017 27810318

[ref108] Happel K. I. Nelson S. (2005). Alcohol, immunosuppression, and the lung. Proc. Am. Thorac. Soc. 2, 428–432. doi: 10.1513/pats.200507-065js 16322595

[ref109] Hayakawa K. Yamaguchi T. Ono D. Suzuki H. Kamiyama J. Taguchi S.. (2020). Two cases of Intrafamilial transmission of community-acquired methicillin-resistant *Staphylococcus aureus* producing both PVL and TSST-1 causing fatal necrotizing pneumonia and Sepsis. Infect Drug Resist 13, 2921–2927. doi: 10.2147/idr.s262123, PMID: 32903848 PMC7445494

[ref110] Hayes A. Lacey J. A. Morris J. M. Davies M. R. Tong S. Y. C. (2020). Restricted sequence variation in *Streptococcus pyogenes* penicillin binding proteins. Msphere 5:e00090. doi: 10.1128/msphere.00090-20 32350098 PMC7193039

[ref111] Hernández-García M. García-Castillo M. Bou G. Cercenado E. Delgado-Valverde M. Oliver A.. (2022). Imipenem-Relebactam susceptibility in Enterobacterales isolates recovered from ICU patients from Spain and Portugal (SUPERIOR and STEP studies). Microbiol. Spectr. 10, e02922–e02927. doi: 10.1128/spectrum.02927-22, PMID: 36043877 PMC9602286

[ref112] Herrera A. L. Huber V. C. Chaussee M. S. (2016). The association between invasive group A streptococcal diseases and viral respiratory tract infections. Front. Microbiol. 7:342. doi: 10.3389/fmicb.2016.00342 27047460 PMC4800185

[ref113] Herrera-Espejo S. del Barrio-Tofiño E. Cebrero-Cangueiro T. López-Causapé C. Álvarez-Marín R. Cisneros J. M.. (2022). Carbapenem combinations for infections caused by Carbapenemase-producing *Pseudomonas aeruginosa*: experimental in vitro and in vivo analysis. Antibiotics 11:1212. doi: 10.3390/antibiotics11091212, PMID: 36139991 PMC9495166

[ref114] Hinrichs C. Wiese-Posselt M. Graf B. Geffers C. Weikert B. Enghard P.. (2022). Successful control of Candida auris transmission in a German COVID-19 intensive care unit. Mycoses 65, 643–649. doi: 10.1111/myc.13443, PMID: 35419847 PMC9115290

[ref115] Hu Z. Yao Y. Chen W. Bian J. Zhao L. Chen L.. (2018). Partial depletion of regulatory T cells enhances host inflammatory response against acute *Pseudomonas aeruginosa* infection after Sepsis. Inflammation 41, 1780–1790. doi: 10.1007/s10753-018-0821-8, PMID: 29956070

[ref116] Hu H. Zhang Y. Zhang P. Wang J. Yuan Q. Shi W.. (2021). Bloodstream infections caused by *Klebsiella pneumoniae* Carbapenemase–Producing *P. aeruginosa* sequence type 463, associated with high mortality rates in China: A retrospective cohort study. Front. Microbiol. 11:756782. doi: 10.3389/fcimb.2021.756782, PMID: 34790589 PMC8592259

[ref117] Huang H. Bai K. Fu Y. Yan J. Li J. (2020). Ecthyma gangrenosum due to *Pseudomonas aeruginosa* sepsis as initial manifestation of X-linked agammaglobulinemia: a case report. BMC Pediatr. 20:540. doi: 10.1186/s12887-020-02436-8 33261572 PMC7704585

[ref118] Huang Y. T. Chen C.-S. Chen H.-A. Hsu H.-S. Liang M.-H. Chang M.-H.. (2020). *Klebsiella pneumoniae* bacteremia revisited: comparison between 2007 and 2017 prospective cohorts at a medical center in Taiwan. J. Inf. Secur. 81, 753–757. doi: 10.1016/j.jinf.2020.08.039, PMID: 32860818

[ref119] Huang H. Y. Lu P.-L. Wang Y.-L. Chen T.-C. Chang K. Lin S.-Y. (2020). Usefulness of EQUAL Candida score for predicting outcomes in patients with candidaemia: a retrospective cohort study. Clin. Microbiol. Infect. 26, 1501–1506. doi: 10.1016/j.cmi.2020.01.029 32036049

[ref120] Hyun M. Noh C. I. Ryu S. Y. Kim H. A. (2018). Changing trends in clinical characteristics and antibiotic susceptibility of *Klebsiella pneumoniae* bacteremia. Kor J Intern Med 33, 595–603. doi: 10.3904/kjim.2015.257 PMC5943641 29117671

[ref122] Imam N. Tempone S. Armstrong P. K. McCann R. Johnson S. Worth L. J.. (2019). Increased incidence of community-associated *Staphylococcus aureus* bloodstream infections in Victoria and Western Australia, 2011–2016. Med. J. Austral. 210, 87–88. doi: 10.5694/mja2.12057, PMID: 30712305

[ref123] Imauven O. Colot J. Couadau E. Moury P.-H. Preault A. Vincent F.. (2022). Paediatric and adult patients from New Caledonia Island admitted to the ICU for community-acquired Panton-valentine leucocidin-producing *Staphylococcus aureus* infections. Sci. Rep. 12:11024. doi: 10.1038/s41598-022-15337-w, PMID: 35773383 PMC9247012

[ref124] Imöhl M. Fitzner C. Perniciaro S., and Linden, M. van der (2017). Epidemiology and distribution of 10 superantigens among invasive *Streptococcus pyogenes* disease in Germany from 2009 to 2014. PLoS One 12,:e0180757. doi: 10.1371/journal.pone.0180757 28719668 PMC5515411

[ref125] Iuliano A. D. Roguski K. M. Chang H. H. Muscatello D. J. Palekar R. Tempia S.. (2018). Estimates of global seasonal influenza-associated respiratory mortality: a modelling study. Lancet 391, 1285–1300. doi: 10.1016/s0140-6736(17)33293-2, PMID: 29248255 PMC5935243

[ref126] Jayakumar J. S. Niyas V. K. M. Arjun R. (2022). Group A streptococcal bacteremia: ten years’ experience from a tertiary Care Center in South India. Ind. J. Critic. Care Med. Peer Review. Off. Publ. Ind. Soc. Critic. Care Med. 26, 1019–1021. doi: 10.5005/jp-journals-10071-24306 PMC9492746 36213703

[ref127] Jean C. Louie J. K. Glaser C. A. Harriman K. Hacker J. K. Aranki F.. (2010). Invasive group A streptococcal infection concurrent with 2009 H1N1 influenza. Clin. Infect. Dis. 50, e59–e62. doi: 10.1086/652291, PMID: 20377405

[ref128] Juan C.-H. Chuang C. Chen C.-H. Li L. Lin Y.-T. (2019). Clinical characteristics, antimicrobial resistance and capsular types of community-acquired, healthcare-associated, and nosocomial *Klebsiella pneumoniae* bacteremia. Antimicrob. Resist. Infect. Control 8:1. doi: 10.1186/s13756-018-0426-x 30622702 PMC6318907

[ref129] Jung I. Y. Jeong S. J. Kim Y. K. Kim H. Y. Song Y. G. Kim J. M.. (2020). A multicenter retrospective analysis of the antifungal susceptibility patterns of Candida species and the predictive factors of mortality in south Korean patients with candidemia. Medicine 99:e19494. doi: 10.1097/md.0000000000019494, PMID: 32176090 PMC7440319

[ref130] Kaneko H. Yanagi Y. Otake S. Sato M. Saito T. Nakaminami H. (2023). The emerging threat of methicillin-resistant *Staphylococcus aureus* (MRSA) clone ST22-PT, carrying both Panton–valentine leucocidin and toxic shock syndrome toxin 1 genes. J. Antimicrob. Chemother. 78, 1023–1027. doi: 10.1093/jac/dkad039 36814074

[ref131] Kang C.-I. Kim S.-H. Park W. B. Lee K.-D. Kim H.-B. Kim E.-C.. (2005). Bloodstream infections caused by antibiotic-resistant gram-negative Bacilli: risk factors for mortality and impact of inappropriate initial antimicrobial therapy on outcome. ASM J. CD 49, 760–766. doi: 10.1128/aac.49.2.760-766.2005, PMID: 15673761 PMC547233

[ref132] Kant S. Pancholi V. (2021). Novel tyrosine kinase-mediated phosphorylation with dual specificity plays a key role in the modulation of *Streptococcus pyogenes* physiology and virulence. Front. Microbiol. 12:689246. doi: 10.3389/fmicb.2021.689246 34950110 PMC8689070

[ref133] Kaper J. B. Nataro J. P. Mobley H. L. T. (2004). Pathogenic *Escherichia coli*. Nat. Rev. Microbiol. 2, 123–140. doi: 10.1038/nrmicro818 15040260

[ref134] Karakonstantis S. Ioannou P. Kritsotakis E. I. (2022). Co-isolates of *Acinetobacter baumannii* complex in polymicrobial infections: a meta-analysis. Access Microbiol. 4:acmi000348. doi: 10.1099/acmi.0.000348 36003364 PMC9394532

[ref135] Kariyawasam R. M. Julien D. A. Jelinski D. C. Larose S. L. Rennert-May E. Conly J. M.. (2022). Antimicrobial resistance (AMR) in COVID-19 patients: a systematic review and meta-analysis (November 2019–June 2021). Antimicrob. Resist. Infect. Control 11:45. doi: 10.1186/s13756-022-01085-z, PMID: 35255988 PMC8899460

[ref136] Kathuria S. Singh P. K. Sharma C. Prakash A. Masih A. Kumar A.. (2015). Multidrug-resistant Candida auris misidentified as Candida haemulonii: characterization by matrix-assisted laser desorption ionization–time of flight mass spectrometry and DNA sequencing and its antifungal susceptibility profile variability by Vitek 2, CLSI broth microdilution, and Etest method. J. Clin. Microbiol. 53, 1823–1830. doi: 10.1128/jcm.00367-15, PMID: 25809970 PMC4432077

[ref137] Kayaaslan B. Eser F. Asilturk D. Oktay Z. Hasanoglu I. Kalem A. K.. (2023). Development and validation of COVID-19 associated candidemia score (CAC-score) in ICU patients. Mycoses 66, 128–137. doi: 10.1111/myc.13531, PMID: 36135336 PMC9537877

[ref138] Kayaaslan B. Eser F. Kaya Kalem A. Bilgic Z. Asilturk D. Hasanoglu I.. (2021). Characteristics of candidemia in COVID-19 patients; increased incidence, earlier occurrence and higher mortality rates compared to non-COVID-19 patients. Mycoses 64, 1083–1091. doi: 10.1111/myc.13332, PMID: 34085319 PMC8242769

[ref139] Khaddour K. Sikora A. Tahir N. Nepomuceno D. Huang T. (2020). Case report: the importance of novel coronavirus disease (COVID-19) and coinfection with other respiratory pathogens in the current pandemic. Am. J. Trop. Med. Hygiene 102, 1208–1209. doi: 10.4269/ajtmh.20-0266 PMC7253125 32314699

[ref140] Kim B. Kim J.-H. Lee Y. (2022). Virulence factors associated with *Escherichia coli* bacteremia and urinary tract infection. Ann. Lab. Med. 42, 203–212. doi: 10.3343/alm.2022.42.2.203 34635614 PMC8548248

[ref141] Kim J. H. Suh J. W. Kim M. J. (2021). Epidemiological trends of Candidemia and the impact of adherence to the Candidemia guideline: six-year single-center experience. J. Fungi 7:275. doi: 10.3390/jof7040275 PMC8067511 33917626

[ref142] Kim S. -H. Yoon Y. K. Kim M. J. Sohn J. W. (2013). Risk factors for and clinical implications of mixed Candida/bacterial bloodstream infections. Clin. Microbiol. Infect. 19, 62–68. doi: 10.1111/j.1469-0691.2012.03906.x 22651822 PMC3563231

[ref143] Kochan T. J. Nozick S. H. Medernach R. L. Cheung B. H. Gatesy S. W. M. Lebrun-Corbin M.. (2022). Genomic surveillance for multidrug-resistant or hypervirulent *Klebsiella pneumoniae* among United States bloodstream isolates. BMC Infect. Dis. 22:603. doi: 10.1186/s12879-022-07558-1, PMID: 35799130 PMC9263067

[ref144] Kufel W. D. Parsels K. A. Blaine B. E. Steele J. M. Mahapatra R. Paolino K. M.. (2023). Vancomycin plus ceftaroline for persistent methicillin‐Resistant*staphylococcus aureus*bacteremia. Pharmacotherapy 43, 15–23. doi: 10.1002/phar.2741 36371648

[ref145] Kung Y.-H. Yeh Y.-C. Kuo K.-C. (2020). Clinical characteristics and predictors of community-acquired *pseudomonas aeruginosa* sepsis and nontyphoidal salmonella sepsis in infants: A matched case–control study. Pediatr. Neonat. 61, 522–528. doi: 10.1016/j.pedneo.2020.05.008 32571671

[ref146] Kutlu M. Sayın-Kutlu S. Alp-Çavuş S. Öztürk Ş. B. Taşbakan M. Özhak B.. (2022). Mortality-associated factors of candidemia: a multi-center prospective cohort in Turkey. Eur. J. Clin. Microbiol. Infect. Dis. 41, 597–607. doi: 10.1007/s10096-021-04394-0, PMID: 35083558

[ref147] Kwiecinski J. M. Horswill A. R. (2020). *Staphylococcus aureus* bloodstream infections: pathogenesis and regulatory mechanisms. Curr. Opin. Microbiol. 53, 51–60. doi: 10.1016/j.mib.2020.02.005 32172183 PMC7244392

[ref148] Kwon Y. J. Won E. J. Jeong S. H. Shin K. S. Shin J. H. Kim Y. R.. (2021). Dynamics and predictors of mortality due to Candidemia caused by different Candida species: comparison of intensive care unit-associated Candidemia (ICUAC) and non-ICUAC. J. Fungi 7:597. doi: 10.3390/jof7080597, PMID: 34436136 PMC8397010

[ref149] Lamba M. Sharma D. Sharma R. Vyas A. Mamoria V. (2021). To study the profile of Candida isolates and antifungal susceptibility pattern of neonatal sepsis in a tertiary care hospital of North India. J. Matern. Fetal Neonat. Med. 34, 2655–2659. doi: 10.1080/14767058.2019.1670799 31581861

[ref150] Lansbury L. Lim B. Baskaran V. Lim W. S. (2020). Co-infections in people with COVID-19: a systematic review and meta-analysis. J. Inf. Secur. 81, 266–275. doi: 10.1016/j.jinf.2020.05.046 PMC7255350 32473235

[ref151] Laux C. Peschel A. Krismer B. (2019). *Staphylococcus aureus* colonization of the human nose and interaction with other microbiome members. Microbiol. Spectr. 7, 1–10. doi: 10.1128/microbiolspec.gpp3-0029-2018 PMC11590430 31004422

[ref152] Lee C. Chen Y. Chen I. Chen F. Chien C. (2020). Impact of biofilm production by Candida species and antifungal therapy on mortality of patients with candidemia. Mycoses 63, 1382–1391. doi: 10.1111/myc.13179 32910518

[ref153] Lee W. J. Hsu J.-F. Chen Y.-N. Wang S.-H. Chu S.-M. Huang H.-R.. (2022). Pediatric Candida bloodstream infections complicated with mixed and subsequent bacteremia: the clinical characteristics and impacts on outcomes. J. Fungi 8:1155. doi: 10.3390/jof8111155, PMID: 36354922 PMC9695890

[ref154] Lee C. M. Kim Y.-J. Jung S.-I. Kim S. E. Park W. B. Choe P. G.. (2022). Different clinical characteristics and impact of carbapenem-resistance on outcomes between Acinetobacter baumannii and *Pseudomonas aeruginosa* bacteraemia: a prospective observational study. Sci. Rep. 12:8527. doi: 10.1038/s41598-022-12482-0, PMID: 35595789 PMC9123196

[ref155] Lee E. H. Lee K. H. Lee S. J. Kim J. Baek Y. J. Ahn J. Y.. (2022). Clinical and microbiological characteristics of and risk factors for bloodstream infections among patients with extracorporeal membrane oxygenation: a single-center retrospective cohort study. Sci. Rep. 12:15059. doi: 10.1038/s41598-022-19405-z, PMID: 36064957 PMC9445101

[ref156] Lee S. Song K.-H. Jung S.-I. Park W. B. Lee S. H. Kim Y.-S.. (2018). Comparative outcomes of cefazolin versus nafcillin for methicillin-susceptible *Staphylococcus aureus* bacteraemia: a prospective multicentre cohort study in Korea. CMI 24, 152–158. doi: 10.1016/j.cmi.2017.07.001, PMID: 28694202

[ref157] Li X. Gu N. Huang T. Y. Zhong F. Peng G. (2023). *Pseudomonas aeruginosa*: A typical biofilm forming pathogen and an emerging but underestimated pathogen in food processing. Front. Microbiol. 13:1114199. doi: 10.3389/fmicb.2022.1114199 36762094 PMC9905436

[ref158] Li M. Yang S. Yao H. Liu Y. Du M. (2023). Retrospective analysis of epidemiology, risk factors, and outcomes of health care-acquired Carbapenem-resistant *Klebsiella pneumoniae* bacteremia in a Chinese tertiary hospital, 2010–2019. Infect. Dis. Ther. 12, 473–485. doi: 10.1007/s40121-022-00732-7 36520329 PMC9925657

[ref159] Liao Y. Hu G.-H. Xu Y.-F. Che J.-P. Luo M. Zhang H.-M.. (2017). Retrospective analysis of fosfomycin combinational therapy for sepsis caused by carbapenem-resistant *Klebsiella pneumoniae*. Exp. Ther. Med. 13, 1003–1010. doi: 10.3892/etm.2017.4046, PMID: 28450933 PMC5403209

[ref160] Liao C.-H. Huang Y.-T. Hsueh P.-R. (2022). Multicenter surveillance of capsular serotypes, virulence genes, and antimicrobial susceptibilities of *Klebsiella pneumoniae* causing bacteremia in Taiwan, 2017–2019. Front. Microbiol. 13:783523. doi: 10.3389/fmicb.2022.783523 35369508 PMC8971976

[ref161] Lin G.-L. McGinley J. P. Drysdale S. B. Pollard A. J. (2018). Epidemiology and immune pathogenesis of viral Sepsis. Front. Immunol. 9:2147. doi: 10.3389/fimmu.2018.02147 30319615 PMC6170629

[ref162] Liu C. Bayer A. Cosgrove S. E. Daum R. S. Fridkin S. K. Gorwitz R. J.. (2011). Clinical practice guidelines by the Infectious Diseases Society of America for the treatment of methicillin-resistant *Staphylococcus aureus* infections in adults and children. Clin. Infect. Dis. 52, e18–e55. doi: 10.1093/cid/ciq146, PMID: 21208910

[ref163] Liu J. Ding H. Zhao M. Tu F. He T. Zhang L.. (2022). Functionalized erythrocyte membrane-coated nanoparticles for the treatment of *Klebsiella pneumoniae*-induced Sepsis. Front. Microbiol. 13:901979. doi: 10.3389/fmicb.2022.901979, PMID: 35783411 PMC9244542

[ref164] Liu Y. du F. L. Liu P. Mei Y. Wan L. Wei D.. (2018). Molecular epidemiology and virulence features of *Staphylococcus aureus* bloodstream isolates in a regional burn Center in China, 2012–2016. Microb. Drug Resist. 24, 1354–1360. doi: 10.1089/mdr.2017.0209, PMID: 29565724

[ref165] Liu F. Zhong L. Zhou F. Zheng C. Zhang K. Cai J.. (2021). Clinical features, strain distribution, antifungal resistance and prognosis of patients with non-albicans Candidemia: A retrospective observational study. Infect Drug Resist 14, 3233–3246. doi: 10.2147/idr.s323583, PMID: 34429621 PMC8380288

[ref166] Lo H.-J. Köhler J. R. DiDomenico B. Loebenberg D. Cacciapuoti A. Fink G. R. (1997). Nonfilamentous *C. albicans* Mutants Are Avirulent. Cells 90, 939–949. doi: 10.1016/s0092-8674(00)80358-x 9298905

[ref167] Loof T. G. Sohail A. Bahgat M. M. Tallam A. Arshad H. Akmatov M. K.. (2018). Early lymphocyte loss and increased granulocyte/lymphocyte ratio predict systemic spread of *Streptococcus pyogenes* in a mouse model of acute skin infection. Front. Microbiol. 8:101. doi: 10.3389/fcimb.2018.00101, PMID: 29707522 PMC5906586

[ref168] Lu B. Fang Y. Fan Y. Chen X. Wang J. Zeng J.. (2017). High prevalence of macrolide-resistance and molecular characterization of *Streptococcus pyogenes* isolates circulating in China from 2009 to 2016. Front. Microbiol. 8:1052. doi: 10.3389/fmicb.2017.01052, PMID: 28642756 PMC5463034

[ref169] Lu S.-L. Omori H. Zhou Y. Lin Y.-S. Liu C.-C. Wu J.-J.. (2022). VEGF-mediated augmentation of Autophagic and lysosomal activity in endothelial cells defends against intracellular *Streptococcus pyogenes*. MBio 13:e0123322. doi: 10.1128/mbio.01233-22, PMID: 35862783 PMC9426552

[ref170] Lv D. Zuo Y. Wang Y. Wang Z. Xu Y. (2022). Predictors of occurrence and 30-Day mortality for co-infection of Carbapenem-resistant Klebsiella pneumoniae and Carbapenem-resistant *Acinetobacter baumannii*. Front Cell Infect Microbiol. 12:919414. doi: 10.3389/fcimb.2022.919414 35795185 PMC9250988

[ref171] Martin R. M. Cao J. Brisse S. Passet V. Wu W. Zhao L.. (2016). Molecular epidemiology of colonizing and infecting isolates of *Klebsiella pneumoniae*. Msphere 1:e00261. doi: 10.1128/msphere.00261-16, PMID: 27777984 PMC5071533

[ref172] Masterson C. H. Murphy E. J. Gonzalez H. Major I. McCarthy S. D. O'Toole D.. (2020). Purified β-glucans from the shiitake mushroom ameliorates antibiotic-resistant *Klebsiella pneumoniae*-induced pulmonary sepsis. Lett. Appl. Microbiol. 71, 405–412. doi: 10.1111/lam.13358, PMID: 32706908

[ref173] Mazzanti S. Brescini L. Morroni G. Orsetti E. Pocognoli A. Donati A.. (2021). Candidemia in intensive care units over nine years at a large Italian university hospital: comparison with other wards. PLoS One 16:e0252165. doi: 10.1371/journal.pone.0252165, PMID: 34038468 PMC8153423

[ref174] McDaniel M. S. Schoeb T. Swords W. E. (2020). Cooperativity between Stenotrophomonas maltophilia and *Pseudomonas aeruginosa* during Polymicrobial airway infections. Infect. Immun. 88, 1–15. doi: 10.1128/iai.00855-19 PMC7093137 31932329

[ref175] McHale T. M. Garciarena C. D. Fagan R. P. Smith S. G. J. Martin-Loches I. Curley G. F.. (2018). Inhibition of vascular endothelial cell leak following *Escherichia coli* attachment in an experimental model of Sepsis. Crit. Care Med. 46, e805–e810. doi: 10.1097/ccm.0000000000003219, PMID: 29782355

[ref176] McNeil J. C. Joseph M. Sommer L. M. Flores A. R. (2022). *Staphylococcus aureus* colonization in healthy children during the first year of the severe acute respiratory syndrome coronavirus 2 pandemic. J. Pediatr. 249, 101–105.e1. doi: 10.1016/j.jpeds.2022.06.025 35772509 PMC9235215

[ref177] Meehan M. Murchan S. Gavin P. J. Drew R. J. Cunney R. (2018). Epidemiology of an upsurge of invasive group A streptococcal infections in Ireland, 2012–2015. J. Inf. Secur. 77, 183–190. doi: 10.1016/j.jinf.2018.05.010 29935196

[ref178] Meyahnwi D. Siraw B. B. Reingold A. (2022). Epidemiologic features, clinical characteristics, and predictors of mortality in patients with candidemia in Alameda County, California; a 2017–2020 retrospective analysis. BMC Infect. Dis. 22:843. doi: 10.1186/s12879-022-07848-8 36371155 PMC9652840

[ref179] Mohr A. Simon M. Joha T. Hanses F. Salzberger B. Hitzenbichler F. (2020). Epidemiology of candidemia and impact of infectious disease consultation on survival and care. Infection 48, 275–284. doi: 10.1007/s15010-020-01393-9 32052287

[ref180] Monecke S. Syed M. A. Khan M. A. Ahmed S. Tabassum S. Gawlik D.. (2020). Genotyping of methicillin-resistant *Staphylococcus aureus* from sepsis patients in Pakistan and detection of antibodies against staphylococcal virulence factors. Eur. J. Clin. Microbiol. Infect. Dis. 39, 85–92. doi: 10.1007/s10096-019-03695-9, PMID: 31482419

[ref181] Morens D. M. Fauci A. S. (2007). The 1918 influenza pandemic: insights for the 21st century. J. Infect. Dis. 195, 1018–1028. doi: 10.1086/511989 17330793

[ref182] Morgado S. Fonseca E. Vicente A. C. (2022). Genomics of *Klebsiella pneumoniae* species complex reveals the circulation of high-risk multidrug-resistant pandemic clones in human, animal, and environmental sources. Microorganisms 10:2281. doi: 10.3390/microorganisms10112281 36422351 PMC9697336

[ref183] Mu A. Klare W. P. Baines S. L. Ignatius Pang C. N. Guérillot R. Harbison-Price N.. (2023). Integrative omics identifies conserved and pathogen-specific responses of sepsis-causing bacteria. Nat. Commun. 14:1530. doi: 10.1038/s41467-023-37200-w, PMID: 36934086 PMC10024524

[ref184] Murray C. J. L. Ikuta K. S. Sharara F. Swetschinski L. Robles Aguilar G. Gray A.. (2022). Global burden of bacterial antimicrobial resistance in 2019: a systematic analysis. Lancet 399, 629–655. doi: 10.1016/s0140-6736(21)02724-0, PMID: 35065702 PMC8841637

[ref185] Naylor N. R. Atun R. Zhu N. Kulasabanathan K. Silva S. Chatterjee A.. (2018). Estimating the burden of antimicrobial resistance: a systematic literature review. Antimicrob. Resist. Infect. Control 7:58. doi: 10.1186/s13756-018-0336-y, PMID: 29713465 PMC5918775

[ref186] Ngamchokwathana C. Chongtrakool P. Waesamaae A. Chayakulkeeree M. (2021). Risk factors and outcomes of non-albicans Candida bloodstream infection in patients with Candidemia at Siriraj hospital—Thailand’s largest National Tertiary Referral Hospital. J. Fungi 7:269. doi: 10.3390/jof7040269 PMC8066724 33916156

[ref187] Nichols K. B. Totsika M. Moriel D. G. Lo A. W. Yang J. Wurpel D. J.. (2016). Molecular characterization of the Vacuolating autotransporter toxin in Uropathogenic *Escherichia coli*. J. Bacteriol. 198, 1487–1498. doi: 10.1128/jb.00791-15, PMID: 26858103 PMC4859599

[ref188] Ohnuma T. Chihara S. Costin B. Treggiari M. Bartz R. Raghunathan K.. (2023). Epidemiology, resistance profiles, and outcomes of bloodstream infections in community-onset Sepsis in the United States. Crit. Care Med. doi: 10.1097/ccm.0000000000005870 (Epub ahead of print). 37276351

[ref189] Okun E. Arumugam T. V. Tang S. Gleichmann M. Albeck M. Sredni B.. (2007). The organotellurium compound ammonium trichloro(dioxoethylene-0,0′) tellurate enhances neuronal survival and improves functional outcome in an ischemic stroke model in mice. J. Neurochem. 102, 1232–1241. doi: 10.1111/j.1471-4159.2007.04615.x, PMID: 17542809

[ref190] Omrani A. S. Koleri J. Ben Abid F. Daghfel J. Odaippurath T. Peediyakkal M. Z.. (2021). Clinical characteristics and risk factors for COVID-19-associated Candidemia. Med. Mycol. 59, 1262–1266. doi: 10.1093/mmy/myab056, PMID: 34625808

[ref191] Osuchowski M. F. Ayala A. Bahrami S. Bauer M. Boros M. Cavaillon J.-M.. (2018). Minimum quality threshold in pre-clinical sepsis studies (MQTiPSS): an international expert consensus initiative for improvement of animal modeling in sepsis. Intensive Care Med. Exp. 6:26. doi: 10.1186/s40635-018-0189-y, PMID: 30112605 PMC6093828

[ref192] Otero-Asman J. R. García-García A. I. Civantos C. Quesada J. M. Llamas M. A. (2019). *Pseudomonas aeruginosa* possesses three distinct systems for sensing and using the host molecule haem. Environ. Microbiol. 21, 4629–4647. doi: 10.1111/1462-2920.14773 31390127

[ref193] Otto N. A. Pereverzeva L. Leopold V. Ramirez-Moral I. Roelofs J. J. T. H. Heijst J. W. J. van. (2021). Hypoxia-inducible factor-1α in macrophages, but not in neutrophils, is important for host defense during *Klebsiella pneumoniae*-induced Pneumosepsis. Mediat. Inflamm. 2021, 1–12. doi: 10.1155/2021/9958281, PMID: 34393650 PMC8360744

[ref194] Pachón-Ibáñez M. E. Labrador-Herrera G. Cebrero-Cangueiro T. Díaz C. Smani Y. Palacio J. P. del. (2018). Efficacy of Colistin and its combination with rifampin in vitro and in experimental models of infection caused by Carbapenemase-producing clinical isolates of *Klebsiella pneumoniae*. Front. Microbiol. 9::912. doi: 10.3389/fmicb.2018.00912, PMID: 29867823 PMC5962653

[ref195] Pappas P. G. Kauffman C. A. Andes D. R. Clancy C. J. Marr K. A. Ostrosky-Zeichner L.. (2016). Clinical practice guideline for the Management of Candidiasis: 2016 update by the Infectious Diseases Society of America. Clin. Infect. Dis. 62, e1–e50. doi: 10.1093/cid/civ933, PMID: 26679628 PMC4725385

[ref196] Park S. Y. Lee J. S. Oh J. Park J.-Y. (2020). Delta neutrophil index as a predictive and prognostic factor for Candidemia patients: a matched case-control study. BMC Infect. Dis. 20:396. doi: 10.1186/s12879-020-05117-0 32503442 PMC7275408

[ref197] Parruti G. Frattari A. Polilli E. Savini V. Sciacca A. Consorte A.. (2019). Cure of recurring *Klebsiella pneumoniae* carbapenemase-producing *Klebsiella pneumoniae* septic shock episodes due to complicated soft tissue infection using a ceftazidime and avibactam-based regimen: a case report. J. Med. Case Rep. 13:20. doi: 10.1186/s13256-018-1934-2, PMID: 30665450 PMC6341597

[ref198] Patricio P. Paiva J. A. Borrego L. M. (2019). Immune response in bacterial and Candida Sepsis. Eur. J. Microbiol. Immunol. 9, 105–113. doi: 10.1556/1886.2019.00011 PMC6945997 31934361

[ref199] Perlee D. Vos A. F. Scicluna B. P. Mancheño P. Rosa O. Dalemans W.. (2019). Human adipose-derived mesenchymal stem cells modify lung immunity and improve antibacterial defense in Pneumosepsis caused by *Klebsiella pneumoniae*. Stem Cells Transl. Med. 8, 785–796. doi: 10.1002/sctm.18-0260, PMID: 31033196 PMC6646807

[ref200] Pfaller M. A. Diekema D. J. Turnidge J. D. Castanheira M. Jones R. N. (2019). Twenty years of the SENTRY antifungal surveillance program: results for Candida species from 1997–2016. Open Forum Infect. Dis. 6, S79–S94. doi: 10.1093/ofid/ofy358 30895218 PMC6419901

[ref201] Pieralli F. Dentali F. Giusti M. Ciarambino T. Mazzone A. Concia E.. (2021). Clinical characteristics, management and outcome of patients with invasive candidiasis hospitalized in internal medicine units: findings from a registry by the Italian scientific society FADOI. Infection 49, 277–285. doi: 10.1007/s15010-020-01535-z, PMID: 33095391

[ref238] Poissy J. Damonti L. Bignon A. Khanna N. von Kietzell M. Boggian K.. (2020). Risk factors for candidemia: a prospective matched case-control study. Crit. Care 24:109. doi: 10.1186/s13054-020-2766-1, PMID: 32188500 PMC7081522

[ref202] Pujol M. Miró J.-M. Shaw E. Aguado J.-M. San-Juan R. Puig-Asensio M.. (2020). Daptomycin plus Fosfomycin versus Daptomycin alone for methicillin-resistant *Staphylococcus aureus* bacteremia and endocarditis. A randomized clinical trial. Clin. Infect. Dis. 72, 1517–1525. doi: 10.1093/cid/ciaa1081, PMID: 32725216 PMC8096235

[ref203] Rajni E. Singh A. Tarai B. Jain K. Shankar R. Pawar K.. (2021). A high frequency ofCandida aurisBlood stream infections in coronavirus disease 2019 patients admitted to intensive care units, northwestern India: a case control study. Open Forum Infect. Dis. 8:ofab452. doi: 10.1093/ofid/ofab452, PMID: 34904116 PMC8522362

[ref204] Rasmussen G. Idosa B. A. Bäckman A. Monecke S. Strålin K. Särndahl E.. (2019). Caspase-1 inflammasome activity in patients with *Staphylococcus aureus* bacteremia. Microbiol. Immunol. 63, 487–499. doi: 10.1111/1348-0421.12738, PMID: 31403210 PMC6916170

[ref205] Recio R. Viedma E. González-Bodí S. Villa J. Orellana M. Á. Mancheño-Losa M.. (2021). Clinical and bacterial characteristics of *Pseudomonas aeruginosa* affecting the outcome of patients with bacteraemic pneumonia. Antimicrobial Agents Annu. 58:106450. doi: 10.1016/j.ijantimicag.2021.106450, PMID: 34644604

[ref206] Recker M. Laabei M. Toleman M. S. Reuter S. Saunderson R. B. Blane B.. (2017). Clonal differences in *Staphylococcus aureus* bacteraemia-associated mortality. Nat. Microbiol. 2, 1381–1388. doi: 10.1038/s41564-017-0001-x, PMID: 28785103

[ref207] Renieris G. Droggiti D.-E. Katrini K. Koufargyris P. Gkavogianni T. Karakike E.. (2021). Host cystathionine-γ lyase derived hydrogen sulfide protects against *Pseudomonas aeruginosa* sepsis. PLoS Pathog. 17:e1009473. doi: 10.1371/journal.ppat.1009473, PMID: 33770141 PMC8051778

[ref208] Rieg S. Joost I. Weiß V. Peyerl-Hoffmann G. Schneider C. Hellmich M.. (2017). Combination antimicrobial therapy in patients with *Staphylococcus aureus* bacteraemia—a post hoc analysis in 964 prospectively evaluated patients. CMI 23, 406.e1–406.e8. doi: 10.1016/j.cmi.2016.08.026, PMID: 27615722

[ref209] Riera F. O. Caeiro J. P. Angiolini S. C. Vigezzi C. Rodriguez E. Icely P. A.. (2022). Invasive candidiasis: update and current challenges in the Management of this Mycosis in South America. Antibiotics 11:877. doi: 10.3390/antibiotics11070877, PMID: 35884131 PMC9312041

[ref210] Ripoli A. Sozio E. Sbrana F. Bertolino G. Pallotto C. Cardinali G.. (2020). Personalized machine learning approach to predict candidemia in medical wards. Infection 48, 749–759. doi: 10.1007/s15010-020-01488-3, PMID: 32740866

[ref211] Ron M. Brosh-Nissimov T. Korenman Z. Treygerman O. Sagi O. Valinsky L.. (2022). Invasive multidrug-resistant emm93.0 *Streptococcus pyogenes* strain harboring a novel Genomic Island, Israel, 2017-2019. Emerg. Infect. Dis. 28, 118–126. doi: 10.3201/eid2801.210733, PMID: 34932442 PMC8714194

[ref212] Rudd K. E. Johnson S. C. Agesa K. M. Shackelford K. A. Tsoi D. Kievlan D. R.. (2020). Global, regional, and national sepsis incidence and mortality, 1990–2017: analysis for the global burden of disease study. Lancet 395, 200–211. doi: 10.1016/s0140-6736(19)32989-7, PMID: 31954465 PMC6970225

[ref213] Said K. B. Alsolami A. Moussa S. Alfouzan F. Bashir A. I. Rashidi M.. (2022). COVID-19 clinical profiles and fatality rates in hospitalized patients reveal case aggravation and selective co-infection by limited gram-negative Bacteria. Environ. Res. Public Health 19:5270. doi: 10.3390/ijerph19095270, PMID: 35564665 PMC9101447

[ref214] Sánchez-Encinales V. Ludwig G. Tamayo E. García-Arenzana J. M. Muñoz-Almagro C. Montes M. (2019). Molecular characterization of *Streptococcus pyogenes* causing invasive disease in pediatric population in Spain A 12-year study. Pediatr. Infect. Dis. J. 38, 1168–1172. doi: 10.1097/inf.0000000000002471 31738331

[ref215] Santos A. P. Gonçalves L. C. Oliveira A. C. C. Queiroz P. H. P. Ito C. R. M. Santos M. O.. (2022). Bacterial co-infection in patients with COVID-19 hospitalized (ICU and not ICU): review and Meta-analysis. Antibiotics 11:894. doi: 10.3390/antibiotics11070894, PMID: 35884147 PMC9312179

[ref216] Satoh K. Makimura K. Hasumi Y. Nishiyama Y. Uchida K. Yamaguchi H. (2009). Candida auris sp. nov., a novel ascomycetous yeast isolated from the external ear canal of an inpatient in a Japanese hospital. Microbiol. Immunol. 53, 41–44. doi: 10.1111/j.1348-0421.2008.00083.x 19161556

[ref217] Schmitz M. Roux X. Huttner B. Pugin J. (2018). Streptococcal toxic shock syndrome in the intensive care unit. Ann. Intensive Care 8:88. doi: 10.1186/s13613-018-0438-y 30225523 PMC6141408

[ref218] Shah P. J. Tran T. Emelogu F. Tariq F. (2021). Aztreonam, ceftazidime/avibactam, and Colistin combination for the Management of Carbapenemase-Producing *Klebsiella Pneumoniae* Bacteremia: A case report. J. Pharm. Pract. 34, 653–657. doi: 10.1177/0897190019882262 31698984

[ref219] Shakoor S. Khan E. Mir F. Malik F. R. Jamil B. (2017). Secular trends of *Streptococcus pyogenes* sepsis in Pakistan and analysis of clinical features in a hospitalized cohort. Trop. Biomed. 34, 648–656. PMID: 33592933

[ref220] Shane A. L. Sánchez P. J. Stoll B. J. (2017). Neonatal sepsis. Lancet 390, 1770–1780. doi: 10.1016/s0140-6736(17)31002-4 28434651

[ref221] Shastri P. S. Shankarnarayan S. A. Oberoi J. Rudramurthy S. M. Wattal C. Chakrabarti A. (2020). Candida auris candidaemia in an intensive care unit – prospective observational study to evaluate epidemiology, risk factors, and outcome. J. Crit. Care 57, 42–48. doi: 10.1016/j.jcrc.2020.01.004 32062286

[ref222] Shorr A. F. Tabak Y. P. Killian A. D. Gupta V. Liu L. Z. Kollef M. H. (2006). Healthcare-associated bloodstream infection&colon; A distinct entity? Insights from a large U.S. database&ast. Crit. Care Med. 34, 2588–2595. doi: 10.1097/01.ccm.0000239121.09533.09 16915117

[ref223] Singer M. Deutschman C. S. Seymour C. W. Shankar-Hari M. Annane D. Bauer M.. (2016). The third international consensus definitions for Sepsis and septic shock (Sepsis-3). JAMA 315, 801–810. doi: 10.1001/jama.2016.0287, PMID: 26903338 PMC4968574

[ref224] Sitkiewicz I. Musser J. (2017). Deletion of atoR from *Streptococcus pyogenes* results in Hypervirulence in a mouse model of Sepsis and is LuxS independent. Pol. J. Microbiol. 66, 17–24. doi: 10.5604/17331331.1234989 29359701

[ref225] Somayaji R. Hantrakun V. Teparrukkul P. Wongsuvan G. Rudd K. E. Day N. P. J.. (2021). Comparative clinical characteristics and outcomes of patients with community acquired bacteremia caused by *Escherichia coli*, Burkholderia pseudomallei and *Staphylococcus aureus*: A prospective observational study (Ubon-sepsis). PLoS Negl. Trop. Dis. 15:e0009704. doi: 10.1371/journal.pntd.0009704, PMID: 34478439 PMC8415581

[ref226] Souli M. Karaiskos I. Masgala A. Galani L. Barmpouti E. Giamarellou H. (2017). Double-carbapenem combination as salvage therapy for untreatable infections by KPC-2-producing *Klebsiella pneumoniae*. Eur. J. Clin. Microbiol. Infect. Dis. 36, 1305–1315. doi: 10.1007/s10096-017-2936-5 28210888

[ref227] Stevens D. L. Bryant A. E. Yan S. (1994). Invasive group A streptococcal infection: new concepts in antibiotic treatment. Int. J. Antimicrob. Agent 4, 297–301. doi: 10.1016/0924-8579(94)90029-9 18611620

[ref228] Stoll B. J. Puopolo K. M. Hansen N. I. Sánchez P. J. Bell E. F. Carlo W. A.. (2020). Early-onset neonatal Sepsis 2015 to 2017, the rise of Escherichia coli, and the need for novel prevention strategies. JAMA Pediatr. 174:e200593. doi: 10.1001/jamapediatrics.2020.0593, PMID: 32364598 PMC7199167

[ref229] Sun X. Dai Y. Tan G. Liu Y. Li N. (2020). Integration analysis of m6A-SNPs and eQTLs associated with Sepsis reveals platelet degranulation and *Staphylococcus aureus* infection are mediated by m6A mRNA methylation. Front. Genet. 11:7. doi: 10.3389/fgene.2020.00007 32174955 PMC7054457

[ref230] Sun J. Uchiyama S. Olson J. Morodomi Y. Cornax I. Ando N.. (2021). Repurposed drugs block toxin-driven platelet clearance by the hepatic Ashwell-Morell receptor to clear *Staphylococcus aureus* bacteremia. Sci. Transl. Med. 13:eabd6737. doi: 10.1126/scitranslmed.abd6737, PMID: 33762439 PMC9121309

[ref231] Tabah A. Buetti N. Staiquly Q. Ruckly S. Akova M. Aslan A. T.. (2023). Epidemiology and outcomes of hospital-acquired bloodstream infections in intensive care unit patients: the EUROBACT-2 international cohort study. Intensive Care Med. 49, 178–190. doi: 10.1007/s00134-022-06944-2, PMID: 36764959 PMC9916499

[ref232] Tacconelli E. Autenrieth I. B. Peschel A. (2017). Fighting the enemy within. Science 355, 689–690. doi: 10.1126/science.aam6372 28209857

[ref233] Tan T. L. Tan-Loh J. Chiew S. C. Lim K. H. Ng W. W. Akmal M.. (2021). Risk factors and outcome of community onset *Pseudomonas aeruginosa* bacteraemia in two Malaysian district specialist hospitals. Med. J. Malays. 76, 820–827. 34806667

[ref234] Tang P.-C. Lee C.-C. Li C.-W. Li M.-C. Ko W.-C. Lee N.-Y. (2017). Time-to-positivity of blood culture: an independent prognostic factor of monomicrobial *Pseudomonas aeruginosa* bacteremia. J. Microbiol. Immunol. Infect. 50, 486–493. doi: 10.1016/j.jmii.2015.08.014 26455486

[ref235] Tang Q. Wang Q. Sun Z. Kang S. Fan Y. Hao Z. (2021). Bergenin monohydrate attenuates inflammatory response via MAPK and NF-κB pathways against Klebsiella pneumonia infection. Front. Pharmacol. 12:651664. doi: 10.3389/fphar.2021.651664 34017253 PMC8129520

[ref236] Tapia P. Gatica S. Cortés-Rivera C. Otero C. Becerra A. Riedel C. A.. (2019). Circulating endothelial cells from septic shock patients convert to fibroblasts are associated with the resuscitation fluid dose and are biomarkers for survival prediction. Crit. Care Med. 47, 942–950. doi: 10.1097/ccm.0000000000003778, PMID: 30998606

[ref237] Teparrukkul P. Hantrakun V. Day N. P. J. West T. E. Limmathurotsakul D. (2017). Management and outcomes of severe dengue patients presenting with sepsis in a tropical country. PLoS One 12:e0176233. doi: 10.1371/journal.pone.0176233 28437459 PMC5402971

[ref239] Thomson T. N. Campbell P. T. Gibney K. B. (2022). The epidemiology of invasive group A streptococcal disease in Victoria, 2007–2017: an analysis of linked datasets. Austral. NZ. J. Publ. Health 46, 878–883. doi: 10.1111/1753-6405.13290 35980150

[ref240] Togawa A. Toh H. Onozawa K. Yoshimura M. Tokushige C. Shimono N.. (2015). Influence of the bacterial phenotypes on the clinical manifestations in *Klebsiella pneumoniae* bacteremia patients: A retrospective cohort study. J. Infect. Chemother. 21, 531–537. doi: 10.1016/j.jiac.2015.04.004, PMID: 26002138

[ref241] Tsay R.-W. Siu L. K. Fung C.-P. Chang F.-Y. (2002). Characteristics of bacteremia between community-acquired and nosocomial *Klebsiella pneumoniae* infection: risk factor for mortality and the impact of capsular serotypes as a herald for community-acquired infection. Arch. Intern. Med. 162, 1021–1027. doi: 10.1001/archinte.162.9.1021 11996612

[ref242] Tumbarello M. Spanu T. Sanguinetti M. Citton R. Montuori E. Leone F.. (2006). Bloodstream infections caused by extended-Spectrum-β-lactamase-Producing*klebsiella pneumoniae*: risk factors, molecular epidemiology, and clinical outcome. ASM J. CD 50, 498–504. doi: 10.1128/aac.50.2.498-504.2006, PMID: 16436702 PMC1366869

[ref243] Turner C. E. (2022). Can group A streptococcus infections be influenced by viruses in the respiratory tract? Lancet Infect. Dis. 23, 142–144. doi: 10.1016/s1473-3099(22)00865-9 36566769

[ref244] Turnidge J. D. Kotsanas D. Munckhof W. Roberts S. Bennett C. M. Nimmo G. R.. (2009). *Staphylococcus aureus* bacteraemia: a major cause of mortality in Australia and New Zealand. Med. J. Austral. 191, 368–373. doi: 10.5694/j.1326-5377.2009.tb02841.x, PMID: 19807625

[ref245] Ulloa E. Uchiyama S. Gillespie R. Nizet V. Sakoulas G. (2021). Ticagrelor increases platelet-mediated *Staphylococcus aureus* killing resulting in clearance of bacteremia. J. Infect. Dis. 224:jiab146. doi: 10.1093/infdis/jiab146 PMC8599881 33966075

[ref246] van Nieuwenhuyse B. van der Linden D. Chatzis O. Lood C. Wagemans J. Lavigne R.. (2022). Bacteriophage-antibiotic combination therapy against extensively drug-resistant *Pseudomonas aeruginosa* infection to allow liver transplantation in a toddler. Nat. Commun. 13:5725. doi: 10.1038/s41467-022-33294-w, PMID: 36175406 PMC9523064

[ref247] Vandendriessche S. de Boeck H. Deplano A. Phoba M.-F. Lunguya O. Falay D.. (2017). Characterisation of *Staphylococcus aureus* isolates from bloodstream infections, Democratic Republic of the Congo. Eur. J. Clin. Microbiol. Infect. Dis. 36, 1163–1171. doi: 10.1007/s10096-017-2904-0, PMID: 28116552

[ref248] Vázquez-Olvera R. Volkow P. Velázquez-Acosta C. Cornejo-Juárez P. (2023). Candida bloodstream infection in patients with cancer: A retrospective analysis of an 11-year period. Rev. Iberoam. Micol. 40, 3–9. doi: 10.1016/j.riam.2022.12.002 36872132

[ref249] Vázquez-Ucha J. C. Arca-Suárez J. Bou G. Beceiro A. (2020). New Carbapenemase inhibitors: clearing the way for the β-lactams. Int. J. Mol. Sci. 21:9308. doi: 10.3390/ijms21239308 33291334 PMC7731173

[ref250] Vilhonen J. Vuopio J. Vahlberg T. Gröndahl-Yli-Hannuksela K. Rantakokko-Jalava K. Oksi J. (2020). Group A streptococcal bacteremias in Southwest Finland 2007–2018: epidemiology and role of infectious diseases consultation in antibiotic treatment selection. Eur. J. Clin. Microbiol. Infect. Dis. 39, 1339–1348. doi: 10.1007/s10096-020-03851-6 32096108 PMC7303095

[ref251] Villalón P. Bárcena M. Medina-Pascual M. J. Garrido N. Pino-Rosa S. Carrasco G.. (2023). National Surveillance of tetracycline, erythromycin, and clindamycin resistance in invasive *Streptococcus pyogenes*: A retrospective study of the situation in Spain, 2007–2020. Antibiotics 12:99. doi: 10.3390/antibiotics12010099, PMID: 36671301 PMC9854882

[ref252] Walker M. J. Barnett T. C. McArthur J. D. Cole J. N. Gillen C. M. Henningham A.. (2014). Disease manifestations and pathogenic mechanisms of group A Streptococcus. Clin. Microbiol. Rev. 27, 264–301. doi: 10.1128/cmr.00101-13, PMID: 24696436 PMC3993104

[ref253] Weiner-Lastinger L. M. Abner S. Edwards J. R. Kallen A. J. Karlsson M. Magill S. S.. (2020). Antimicrobial-resistant pathogens associated with adult healthcare-associated infections: summary of data reported to the National Healthcare Safety Network, 2015–2017. Infect. Control. 41, 1–18. doi: 10.1017/ice.2019.296, PMID: 31767041 PMC8276252

[ref254] Wesolek J. L. McNorton K. Delgado G. Giuliano C. A. (2018). Effect of vancomycin initial dosing on time to systemic inflammatory response syndrome resolution in patients with methicillin-resistant *Staphylococcus aureus* bacteremia. J. Chemother. 30, 101–106. doi: 10.1080/1120009x.2017.1389807 29065784

[ref255] World Health Organization (WHO) (2017). Improving the prevention, diagnosis and clinical management of sepsis. Available at: https://apps.who.int/gb/ebwha/pdf_files/WHA70/A70_13-en.pdf

[ref256] World Health Organization (WHO) (2022a). Global antimicrobial resistance and use surveillance system (GLASS) report 2022. Available at: https://www.who.int/publications/i/item/9789240062702

[ref257] World Health Organization (WHO) (2022b). WHO fungal priority pathogens list to guide research, development and public health action. Available at: https://www.who.int/publications/i/item/9789240060241

[ref258] Wu H. Chen H. Wang J. Yin S. Huang J. Wang Z.. (2021). Identification of key genes associated with sepsis patients infected by *staphylococcus aureus* through weighted gene co-expression network analysis. Am. J. Transl. Res. 13, 13579–13589. PMID: 35035698 PMC8748107

[ref259] Wu Y. M. Huang P.-Y. Cheng Y.-C. Lee C.-H. Hsu M.-C. Lu J.-J.. (2021). Enhanced virulence of *Candida albicans* by *Staphylococcus aureus*: evidence in clinical bloodstream infections and infected zebrafish embryos. J. Fungi 7:1099. doi: 10.3390/jof7121099, PMID: 34947081 PMC8706905

[ref260] Wu X. Shi Q. Shen S. Huang C. Wu H. (2021). Clinical and bacterial characteristics of *Klebsiella pneumoniae* affecting 30-Day mortality in patients with bloodstream infection. Front Cell Infect Microbiol. 11:688989. doi: 10.3389/fcimb.2021.688989 34604103 PMC8482843

[ref261] Wu Z. Tian Y. Alam H. B. Li P. Duan X. Williams A. M.. (2020). Peptidylarginine deiminases 2 mediates Caspase-1-associated lethality in *Pseudomonas aeruginosa* pneumonia-induced Sepsis. J. Infect. Dis. 223, 1093–1102. doi: 10.1093/infdis/jiaa475, PMID: 32729925

[ref262] Wu T. Xu F. Su C. Li H. Lv N. Liu Y.. (2020). Alterations in the gut microbiome and Cecal metabolome during *Klebsiella pneumoniae*-induced Pneumosepsis. Front. Immunol. 11:1331. doi: 10.3389/fimmu.2020.01331, PMID: 32849494 PMC7411141

[ref263] Wurster S. Albert N. D. Kontoyiannis D. P. (2022). Candida auris bloodstream infection induces upregulation of the PD-1/PD-L1 immune checkpoint pathway in an immunocompetent mouse model. Msphere 7, e00817–e00821. doi: 10.1128/msphere.00817-21 35224979 PMC9044930

[ref264] Xiong W. Perna A. Jacob I. B. Lundgren B. R. Wang G. (2022). The enhancer-binding protein MifR, an essential regulator of α-ketoglutarate transport, is required for full virulence of *Pseudomonas aeruginosa* PAO1 in a mouse model of pneumonia. Infect. Immun. 90, e00122–e00136. doi: 10.1128/iai.00136-22 PMC9584295 36125307

[ref265] Yang T. Y. Hsieh Y.-J. Kao L.-T. Liu G. H. Lian S.-H. Wang L.-C.. (2022). Activities of imipenem-relebactam combination against carbapenem-nonsusceptible Enterobacteriaceae in Taiwan. J. Microbiol. Immunol. Infect. 55, 86–94. doi: 10.1016/j.jmii.2021.02.001, PMID: 33678555

[ref266] Yang T.-Y. Kao H.-Y. Lu P.-L. Chen P.-Y. Wang S.-C. Wang L.-C.. (2021). Evaluation of the Organotellurium compound AS101 for treating Colistin- and Carbapenem-resistant *Klebsiella pneumoniae*. Pharmaceuticals 14:795. doi: 10.3390/ph14080795, PMID: 34451891 PMC8400984

[ref267] Yang F. Zhou Y. Chen P. Cai Z. Yue Z. Jin Y.. (2022). High-level expression of cell-surface signaling system Hxu enhances *Pseudomonas aeruginosa* bloodstream infection. Infect. Immun. 90:e0032922. doi: 10.1128/iai.00329-22, PMID: 36169312 PMC9584290

[ref268] Yi Y.-H. Wang J.-L. Yin W.-J. Xu W.-H. (2021). Vancomycin or Daptomycin plus a β-lactam versus vancomycin or Daptomycin alone for methicillin-resistant *Staphylococcus aureus* bloodstream infections: A systematic review and Meta-analysis. Microb. Drug Resist. 27, 1044–1056. doi: 10.1089/mdr.2020.0350 33728980

[ref269] Yoo J. R. Shin B. R. Jo S. Heo S. T. (2020). Evaluation of the early fluconazole treatment of candidemia protocol with automated short message service alerts: a before-and-after study. Kor J Intern Med 36, 699–705. doi: 10.3904/kjim.2019.259 PMC8137413 32640779

[ref270] Zahedi bialvaei A. Rahbar M. Hamidi-Farahani R. Asgari A. Esmailkhani A. Mardani dashti Y.. (2021a). Expression of RND efflux pumps mediated antibiotic resistance in *Pseudomonas aeruginosa* clinical strains. Microb. Pathog. 153:104789. doi: 10.1016/j.micpath.2021.104789, PMID: 33556480

[ref271] Zahedi bialvaei A. Razavi S. Notash Haghighat F. Hemmati A. Akhavan M. M. Jeddi-Tehrani M.. (2021b). Monoclonal antibody directed to the PilQ -PilA DSL region in *Pseudomonas aeruginosa* improves survival of infected mice with antibiotic combination. Microb. Pathog. 158:105060. doi: 10.1016/j.micpath.2021.105060, PMID: 34153421

[ref272] Zhang W. Song X. Wu H. Zheng R. (2020). Epidemiology, species distribution, and predictive factors for mortality of candidemia in adult surgical patients. BMC Infect. Dis. 20:506. doi: 10.1186/s12879-020-05238-6 32660641 PMC7359486

[ref273] Zhao R. Wang X. Wang X. du B. Xu K. Zhang F.. (2022). Molecular characterization and virulence gene profiling of methicillin-resistant *Staphylococcus aureus* associated with bloodstream infections in southern China. Front. Microbiol. 13:1008052. doi: 10.3389/fmicb.2022.1008052, PMID: 36325019 PMC9618618

[ref274] Zheng G. Zhang J. Wang B. Cai J. Wang L. Hou K.. (2021). Ceftazidime-avibactam in combination with in vitro non-susceptible antimicrobials versus ceftazidime-avibactam in monotherapy in critically ill patients with Carbapenem-resistant *Klebsiella Pneumoniae* infection: A retrospective cohort study. Infect. Dis. Ther. 10, 1699–1713. doi: 10.1007/s40121-021-00479-7, PMID: 34241831 PMC8322179

[ref275] Zhong L. Dong Z. Liu F. Li H. Tang K. Zheng C.. (2022). Incidence, clinical characteristics, risk factors and outcomes of patients with mixed Candida/bacterial bloodstream infections: a retrospective study. ACMA 21:45. doi: 10.1186/s12941-022-00538-y, PMID: 36320023 PMC9628097

[ref276] Zhong L. Zhang S. Tang K. Zhou F. Zheng C. Zhang K.. (2020). Clinical characteristics, risk factors and outcomes of mixed *Candida albicans*/bacterial bloodstream infections. BMC Infect. Dis. 20:810. doi: 10.1186/s12879-020-05536-z, PMID: 33158426 PMC7648279

[ref277] Zhou W. Jin Y. Zhou Y. Wang Y. Xiong L. Luo Q.. (2021). Comparative genomic analysis provides insights into the evolution and genetic diversity of community-genotype sequence type 72 *Staphylococcus aureus* isolates. Msystems 6:e0098621. doi: 10.1128/msystems.00986-21, PMID: 34491085 PMC8547429

